# Supplementary material for: Normalization and de-noising of single-cell Hi-C data with BandNorm and scVI-3D
Source: Genome Biol. 2022 Oct 17;23:222. doi: 10.1186/s13059-022-02774-z (PMC9575231; doi:10.1186/s13059-022-02774-z)
Supplement: Supplementary file 1 — Additional file 1. Supplementary figures [81–83]. [file 13059_2022_2774_MOESM1_ESM.pdf]

Supplementary materials for "Normalization and de-noising of single-cell Hi-C data with BandNorm and scVI-3D"

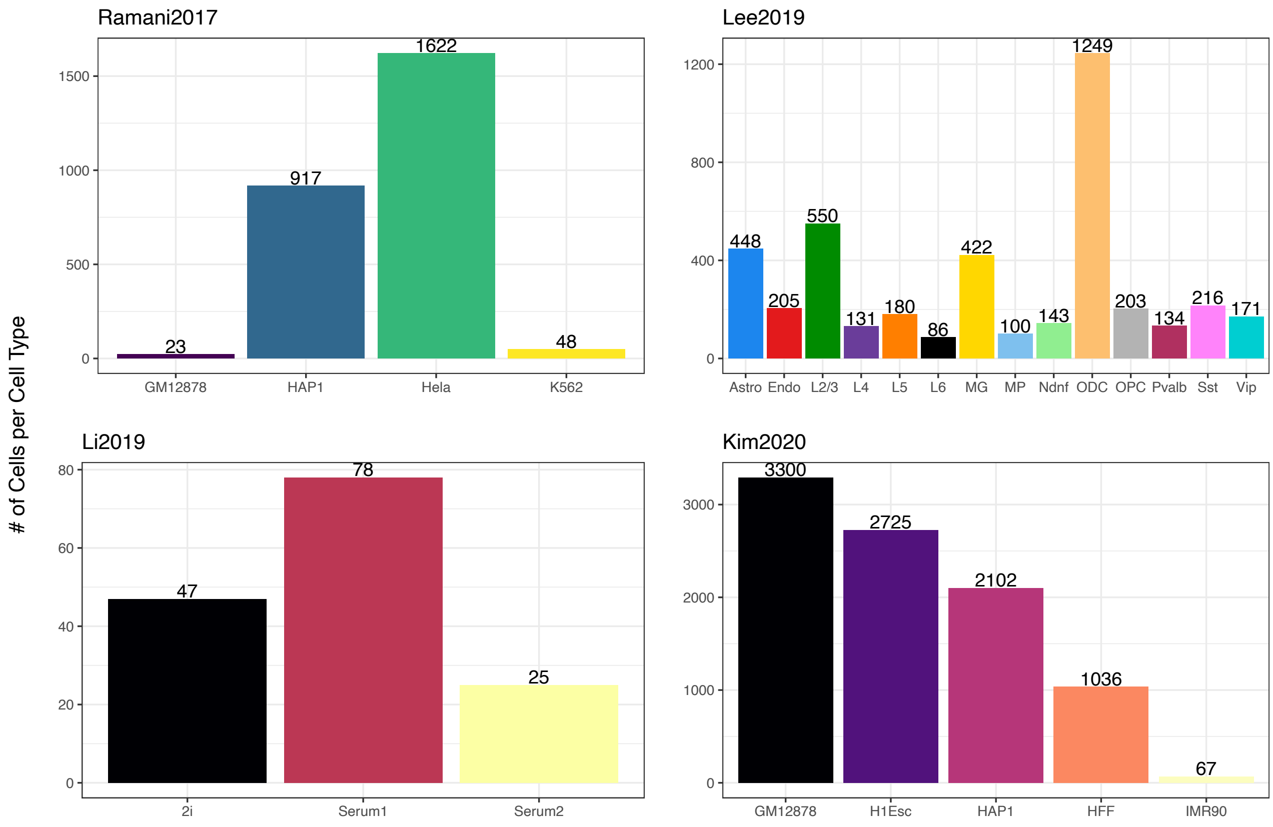

Fig. S1 *Number of valid cells for each cell type in the* `Ramani2017`, `Lee2019`, `Li2019`, and `Kim2020` data sets. Details of cell filtering are available in the Methods.

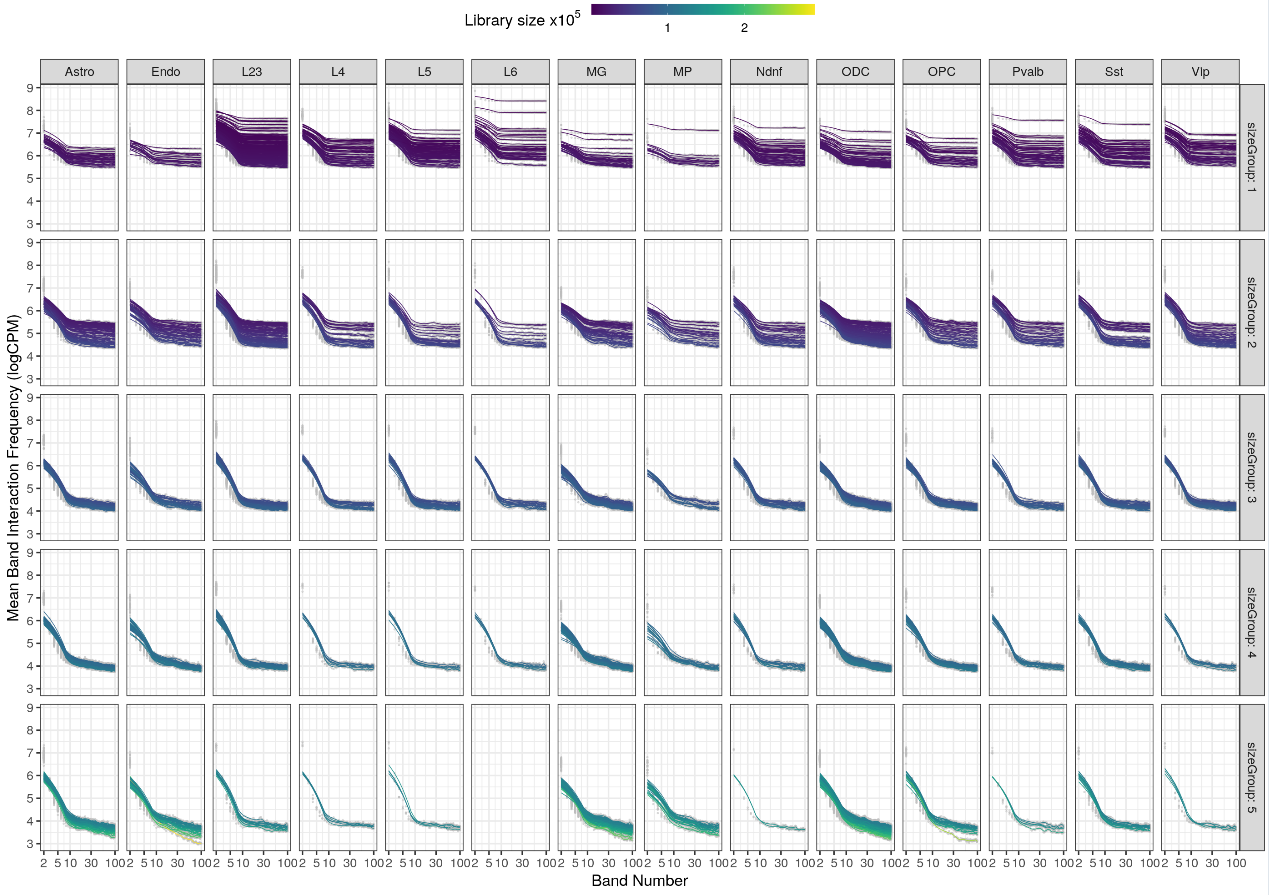

Fig. S2 *Genomic distance effect at different library size levels.* Band effect on the mean band interaction frequencies as a function of library size and cell type for the Lee2019 data set with 14 cell types. Each line depicts the lowess smoothed mean of the interaction frequencies (in log counts per million) with respect to the genomic distance, i.e., the band number, from individual cells.

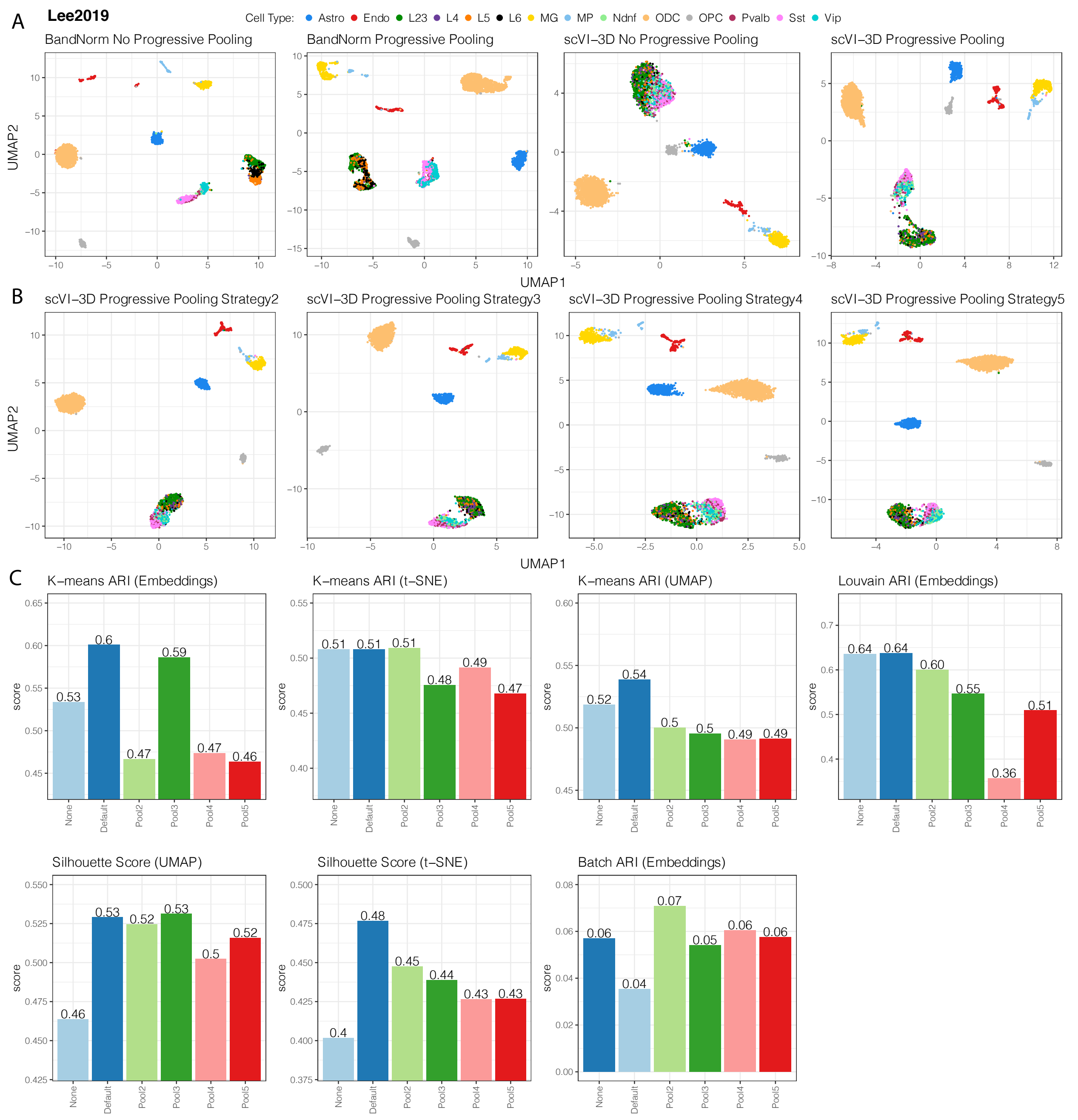

Fig. S3 *Progressive pooling improves cell type separation while maintaining low impact from the batch biases for scVI-3D on* `Lee2019` *data set.* **A-B.** Progressive pooling strategies that combine bands farther off-diagonals are defined in **Methods** section. UMAP visualizations compare the BandNorm and scVI-3D cell type separation performance with or without a progressive pooling strategy. **C.** Quantitative assessment using ARI and Silhouette scores to measure the cell type separation and batch effect on the latent embeddings.

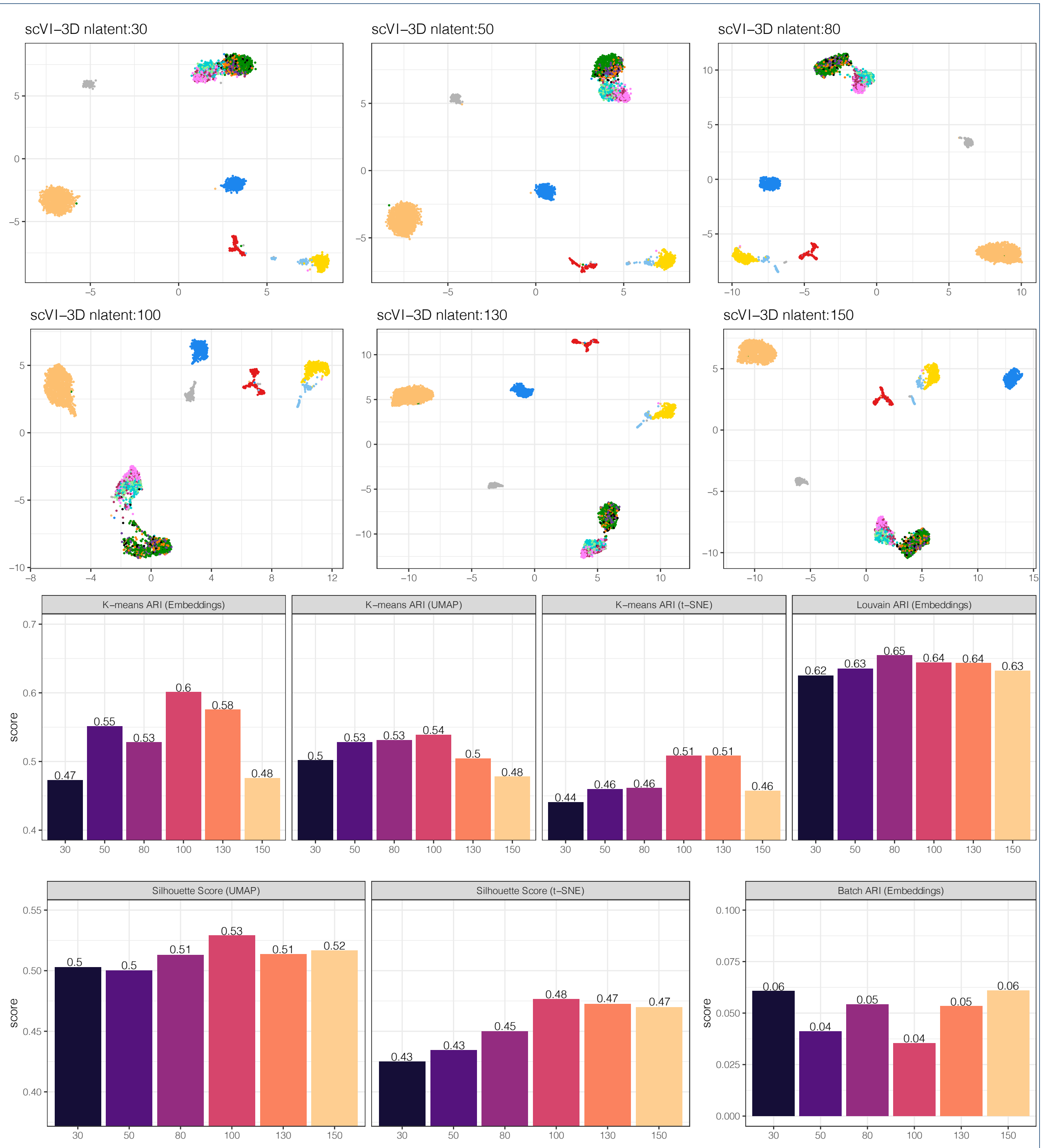

Fig. S4 *Investigation for a proper latent variable dimension of scVI-3D normalization and its impact on the cell type separation of* `Lee2019` *data set.* UMAP visualization and quantitative metrics evaluation on the performance of scVI-3D normalization using a different number of latent variables: 30, 50, 80, 100, 130, and 150. Quantitative assessment includes ARI and Silhouette scores evaluations on full latent embeddings and UMAP or t-SNE dimension reduction embeddings. The batch effect is measured by ARI on the full latent embeddings.

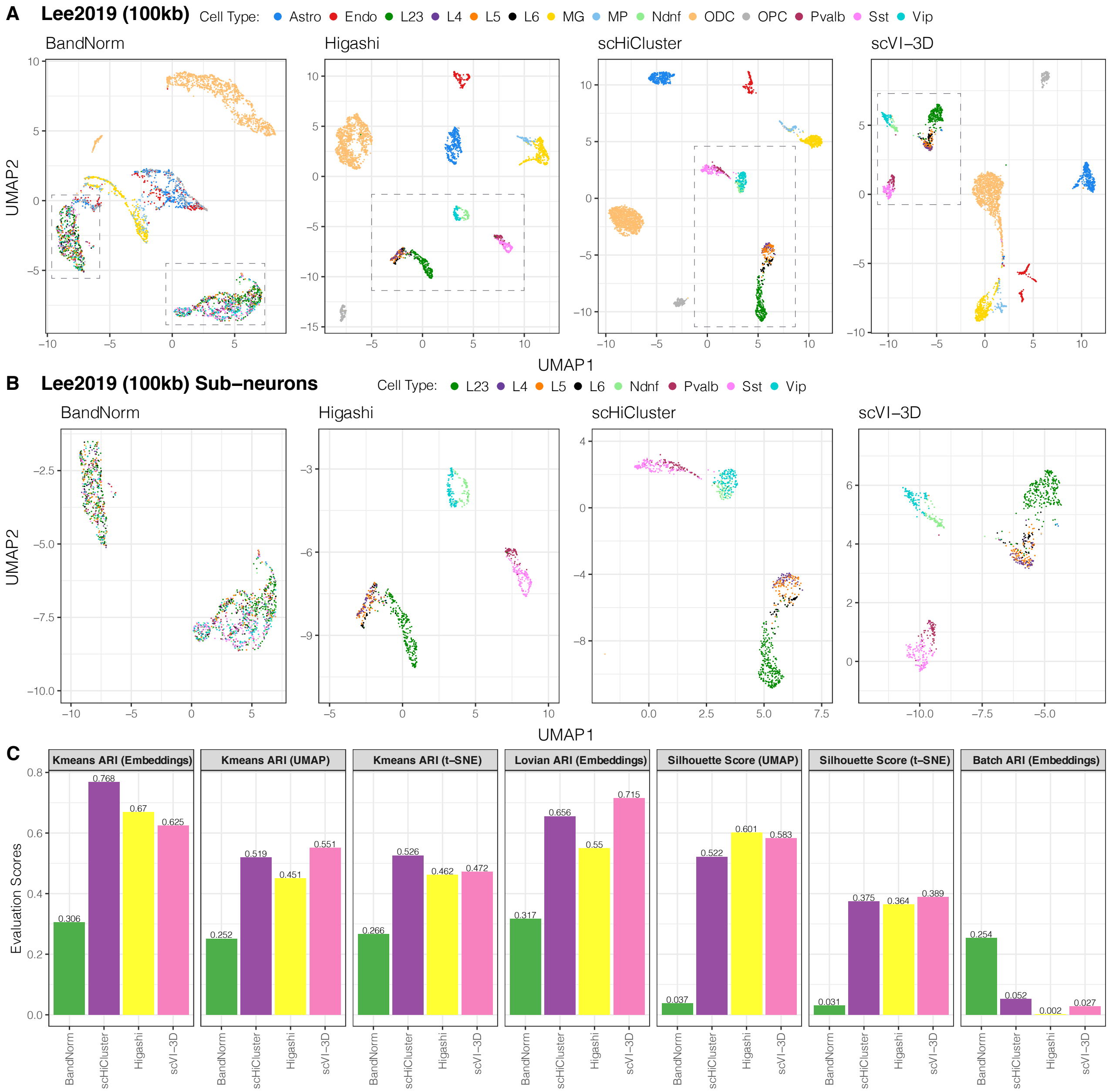

Fig. S5 *Benchmarking scHi-C normalization and de-noising methods for cell-type separation on Lee2019 data set of resolution 100kb.* **A.** Comparison of the scHi-C data normalization and de-noising methods on Lee2019 data set binned at 100kb resolution. Excitatory neuronal subtypes (L2/3, L4, L5, L6) and inhibitory cells (Ndnf, Vip, Pvalb, and Sst) are highlighted by black squares with a zoom-in visualization in **B**. **C.** Quantitative assessment of the cell type separation includes ARI and Silhouette scores on full latent embeddings and UMAP or t-SNE dimension reduction embeddings. The batch effect is evaluated by the ARI on the full latent embeddings.

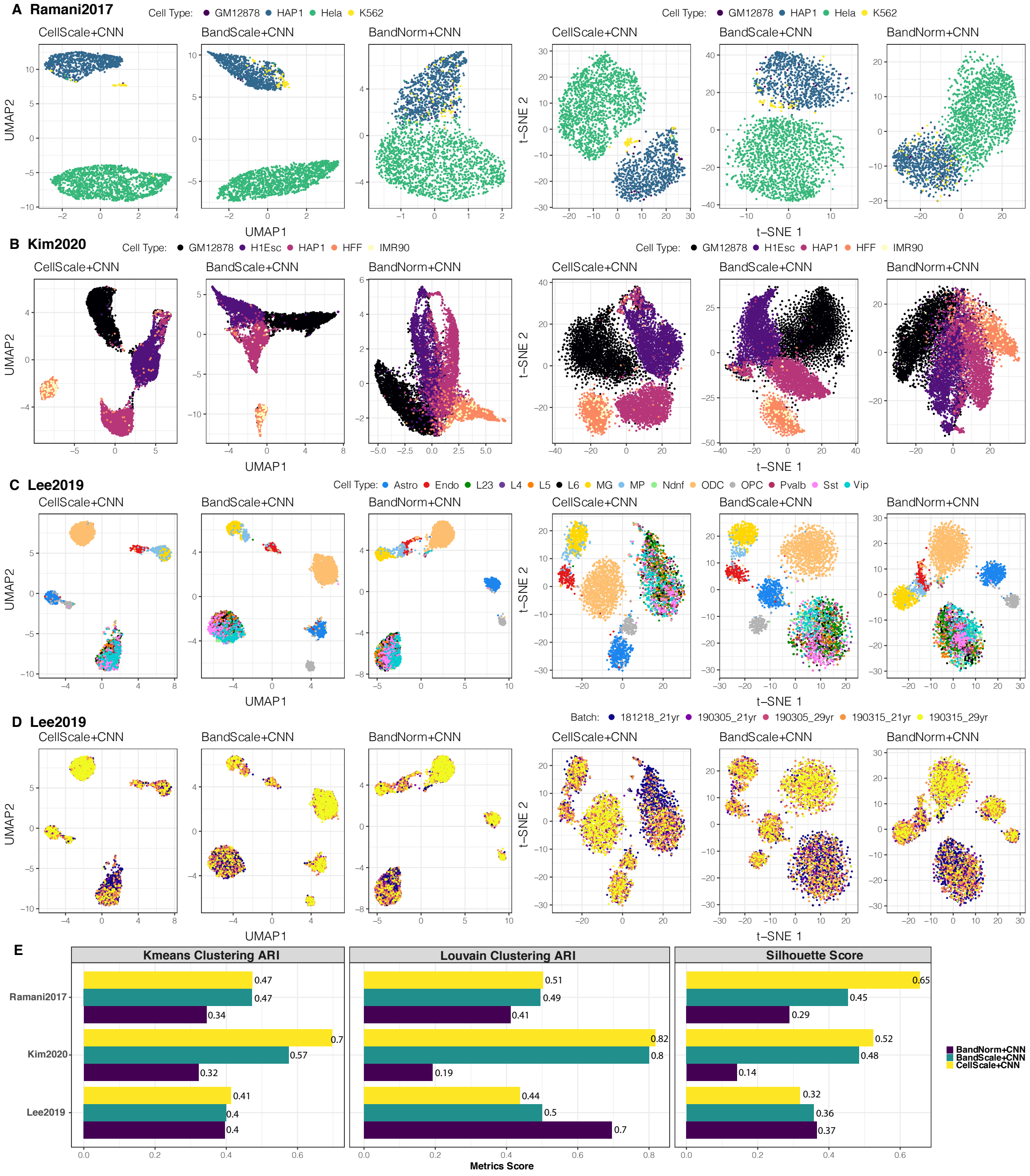

Fig. S6 *Performance of the cell type separation and batch effect removal comparing three scHi-C scaling methods coupled with Convolutional Neural Network (CNN).* Cell type separation performance comparing the CellScale+CNN, BandScale+CNN and BandNorm+CNN on `Ramani2017` (**A**), `Kim2020` (**B**), and `Lee2019` (**C**) data sets. **D.** Harmony was leveraged to remove batch effect of three CNN normalization variants on `Lee2019` data set. For **A**-**D**, left panels are dimension reduction visualization by UMAP and right panels by t-SNE. **E.** ARI and Silhouette scores evaluations of the cell type separation.

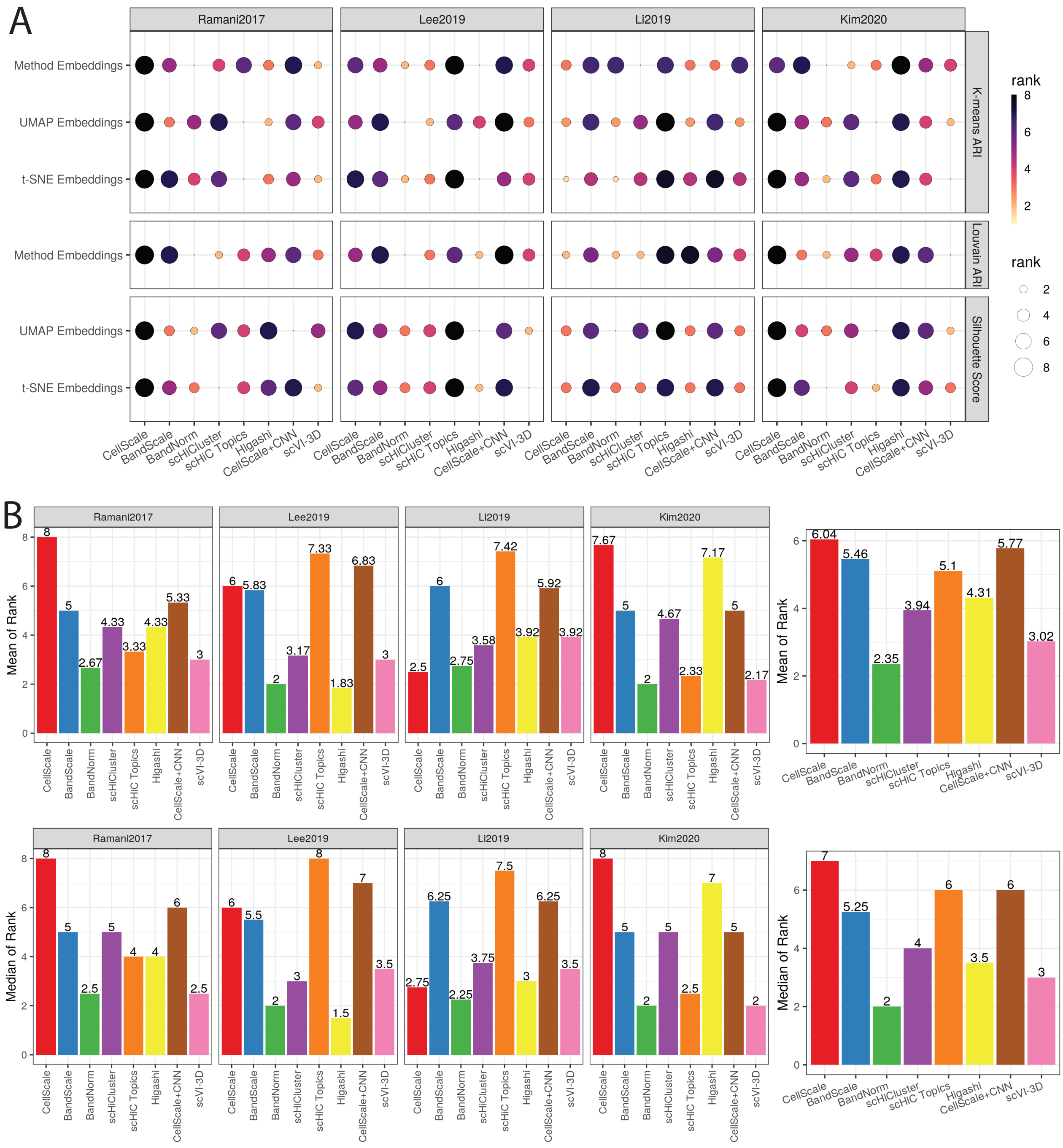

Fig. S7 *Performance ranking of the scHi-C normalization and de-noising methods.* **a.** Rank of the eight scHi-C analysis methods, CellScale, BandScale, BandNorm, scHiCluster, scHiC Topics, Higashi, CellScale + CNN, and scVI-3D, across the six evaluation metrics and four benchmark data sets. **b.** Median (first row) and mean (second row) ranks of the scHi-C methods across the six evaluation metrics for each of the four data sets (left panel) and average over four data sets (right panel).

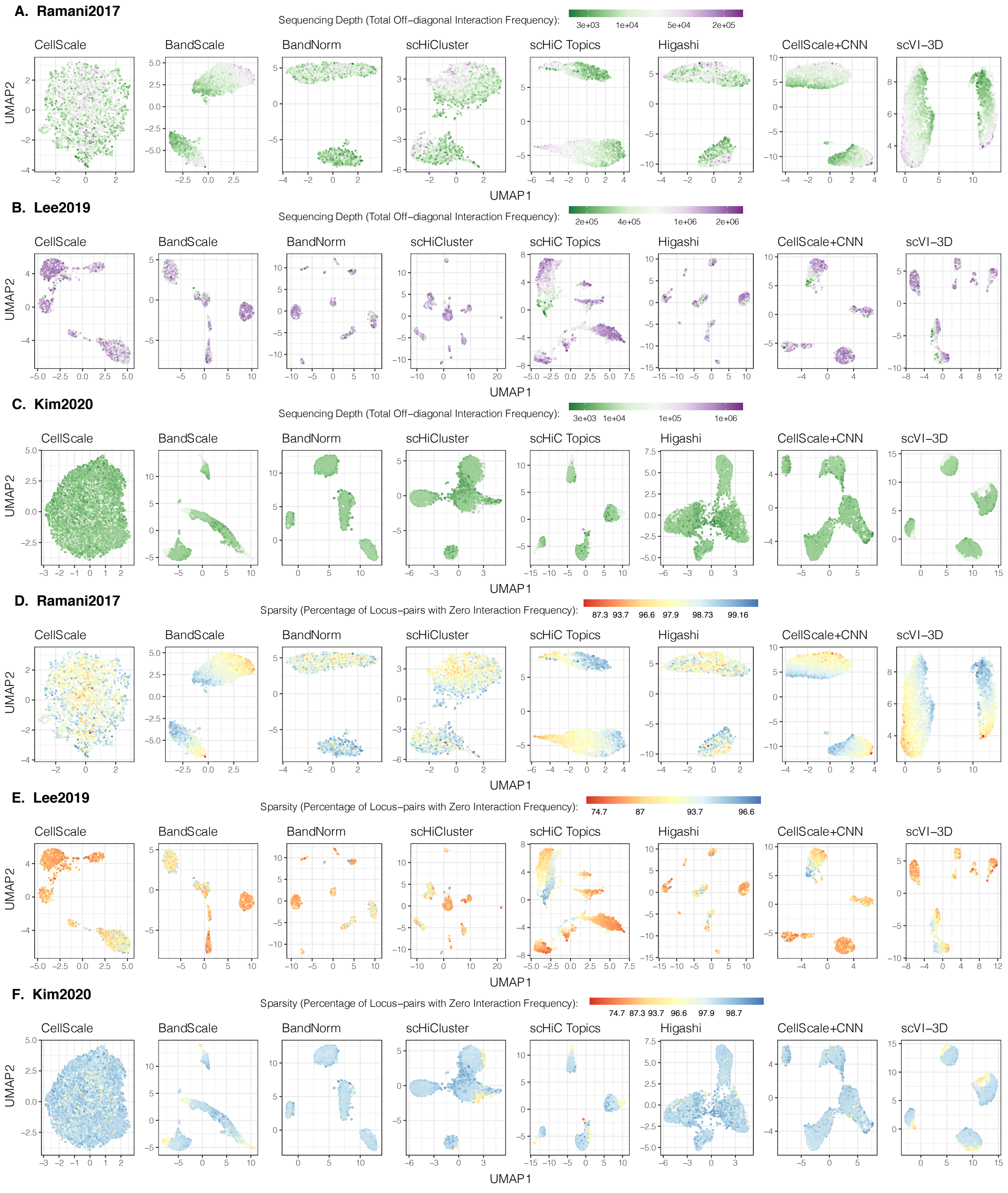

Fig. S8 *Impact of sequencing depth and sparsity on cell type separation.* Sequencing depth for `Ramani2017` (**A**), `Lee2019` (**B**) and `Kim2020` (**C**) data sets are computed as the total interaction frequencies in the upper triangular of the contact matrices. The results are displayed using scatter plots of the two UMAP coordinates. Color shading depicts the sequencing depths of the cells. Sparsity for `Ramani2017` (**D**), `Lee2019` (**E**) and `Kim2020` (**F**) data sets is defined as the percentage of locus-pairs with zero interaction frequency in the upper triangular of the contact matrix. The results are displayed using scatter plots of the two UMAP coordinates. Color shading depicts the sparsity of the cells.

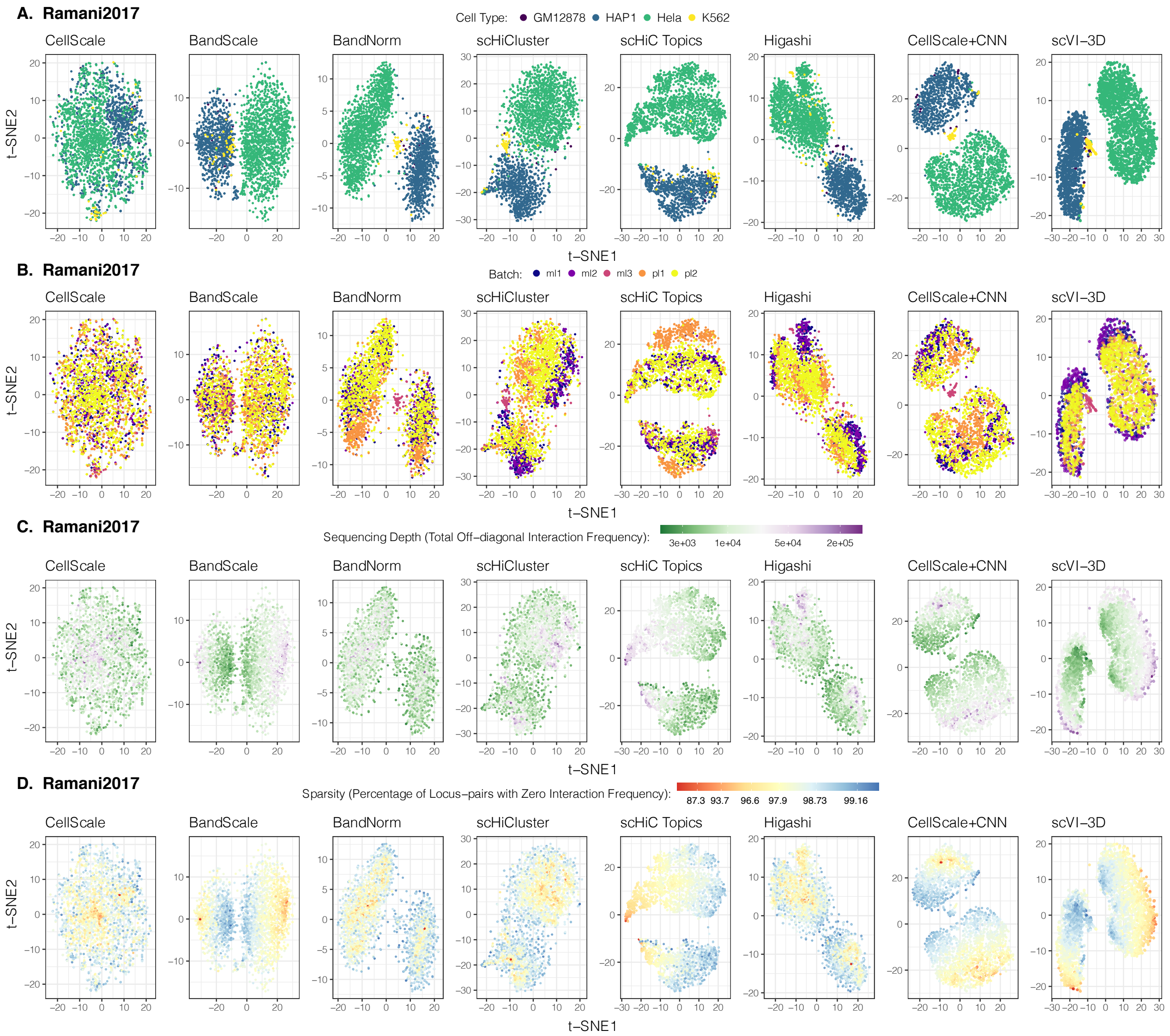

Fig. S9 *t-SNE visualization of the low-dimensional embeddings of the* `Ramani2017` *data set.* Cell type separation performance (**A**), and the impact of batch effect (**B**), sequencing depth (**C**) and sparsity (**D**) for eight scHi-C normalization and de-noising methods. The results are displayed using scatter plots of the two t-SNE coordinates.

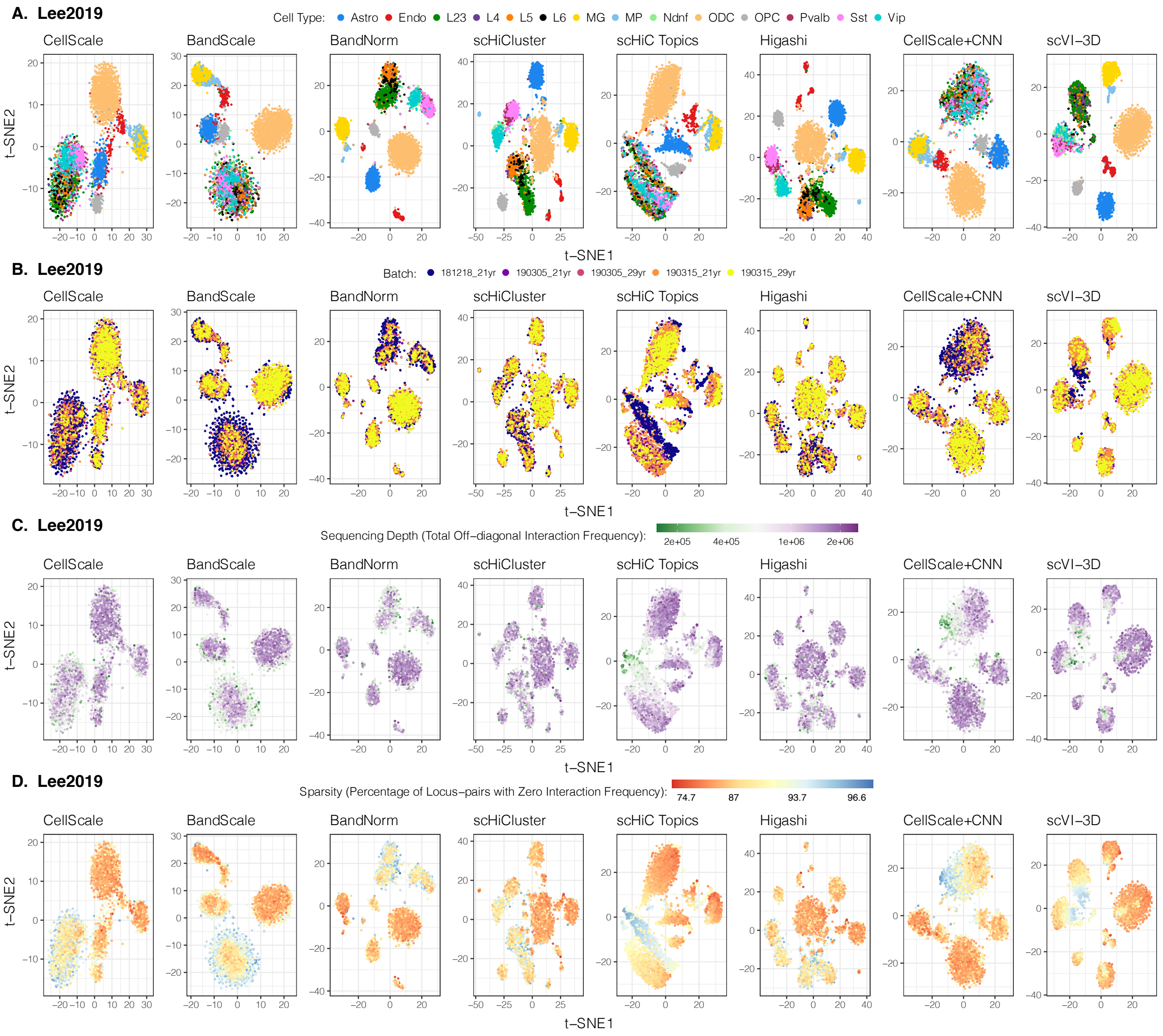

Fig. S10 *t-SNE visualization of the low-dimensional embeddings of the* `Lee2019` *data set.* Cell type separation performance (**a**), and the impact of batch effect (**b**), sequencing depth (**c**) and sparsity (**d**) for the scHi-C normalization and de-noising methods. The results are displayed using scatter plots of the two t-SNE coordinates.

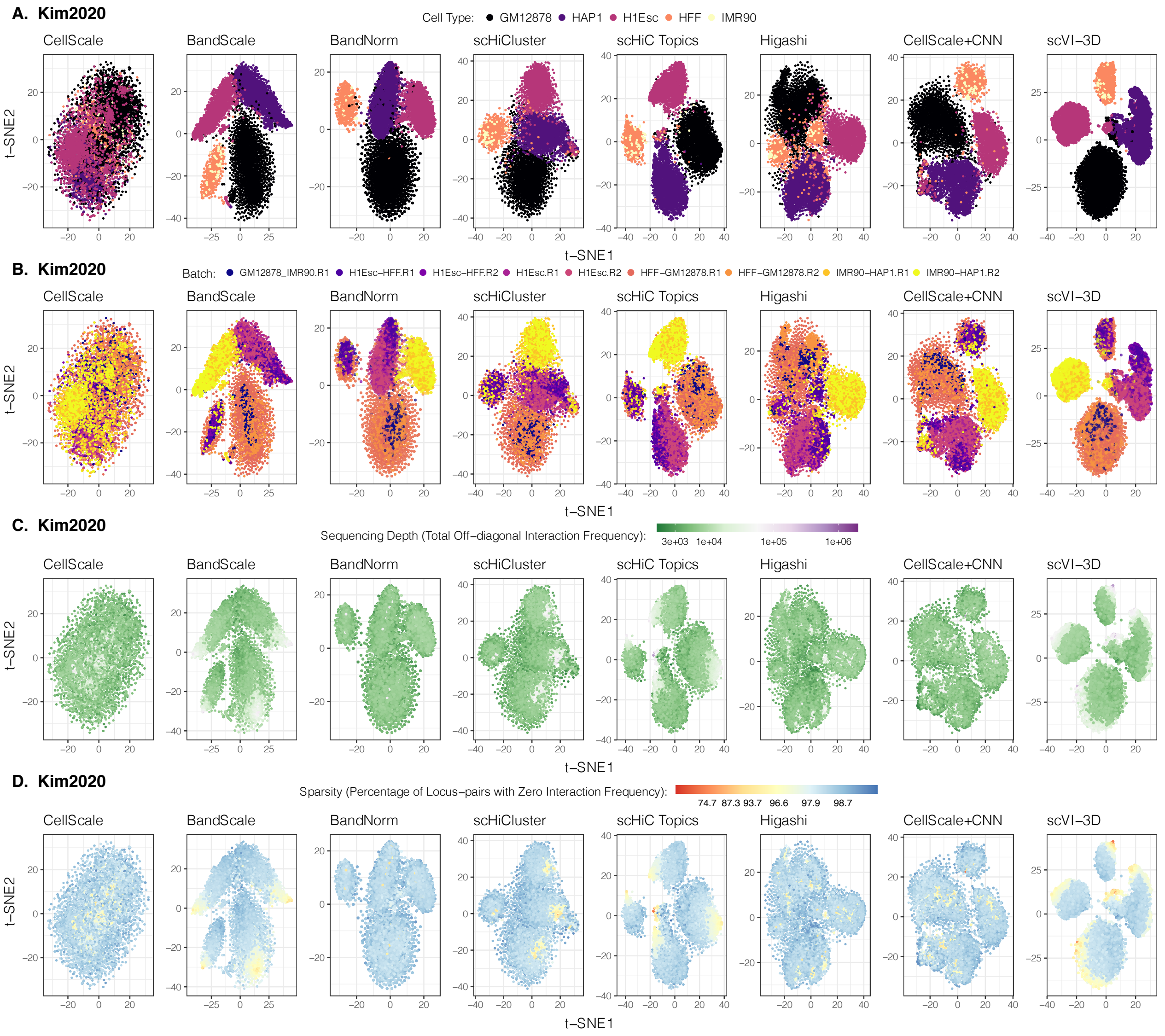

Fig. S11 *t-SNE visualization of the low-dimensional embeddings of the Kim2020 data set.* Cell type separation performance (**a**), and the impact of batch effect (**b**), sequencing depth (**c**) and sparsity (**d**) for the scHi-C normalization and de-noising methods. The results are displayed using scatter plots of the two t-SNE coordinates.

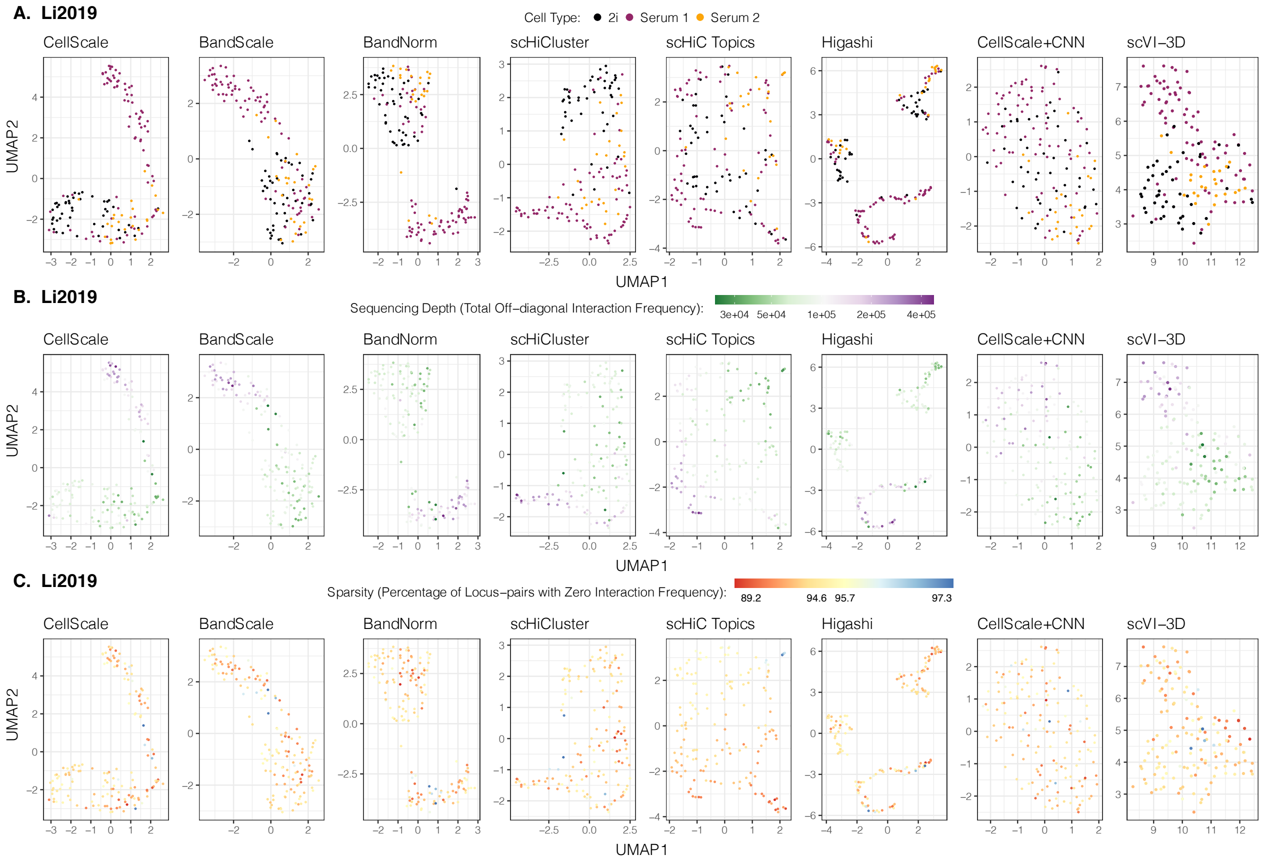

Fig. S12 *UMAP visualization of the low-dimensional embeddings of the Li2019 data set.* Cell type separation performance (**a**), sequencing depth (**b**) and sparsity (**c**) for the scHi-C normalization and de-noising methods. The results are displayed using scatter plots of the two UMAP coordinates.

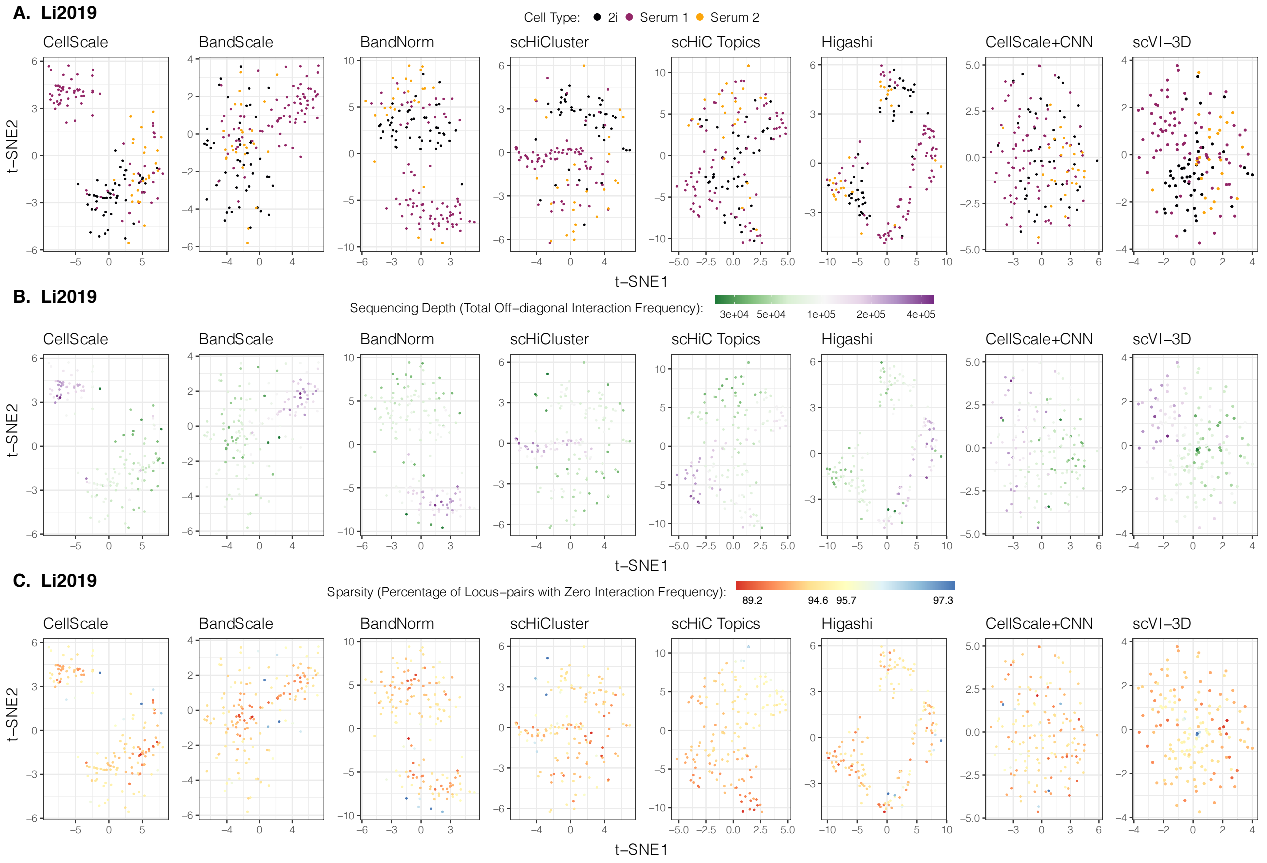

Fig. S13 *t-SNE visualization of the low-dimensional embeddings of the* Li2019 *data set.* Cell type separation performance (**a**), sequencing depth (**b**) and sparsity (**c**) for the scHi-C normalization and de-noising methods. The results are displayed using scatter plots of the two t-SNE coordinates.

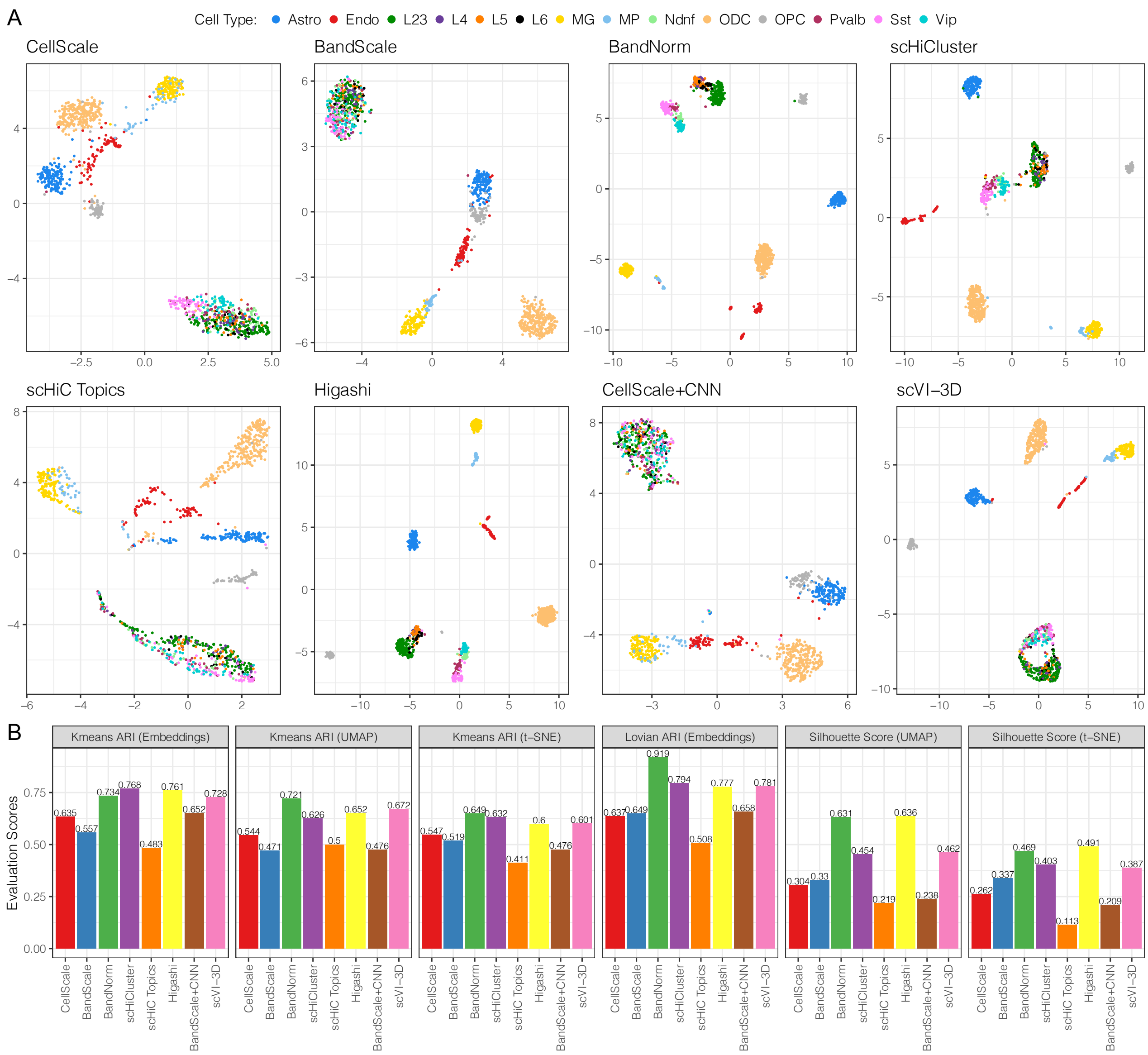

Fig. S14 *Benchmarking scHi-C normalization and de-noising methods for cell-type separation in a batch-free context.* Comparison of the scHi-C data normalization and de-noising methods on one batch with the largest sequencing depth (i.e., 190315_29r) of Lee2019 data set. This provides batch-free context to benchmark scHi-C normalization methods. The results are displayed using scatter plots of the two UMAP coordinates. The colors correspond to cell types. Quantitative assessment of the cell type separation includes ARI and Silhouette scores on full latent embeddings and UMAP or t-SNE dimension reduction embeddings.

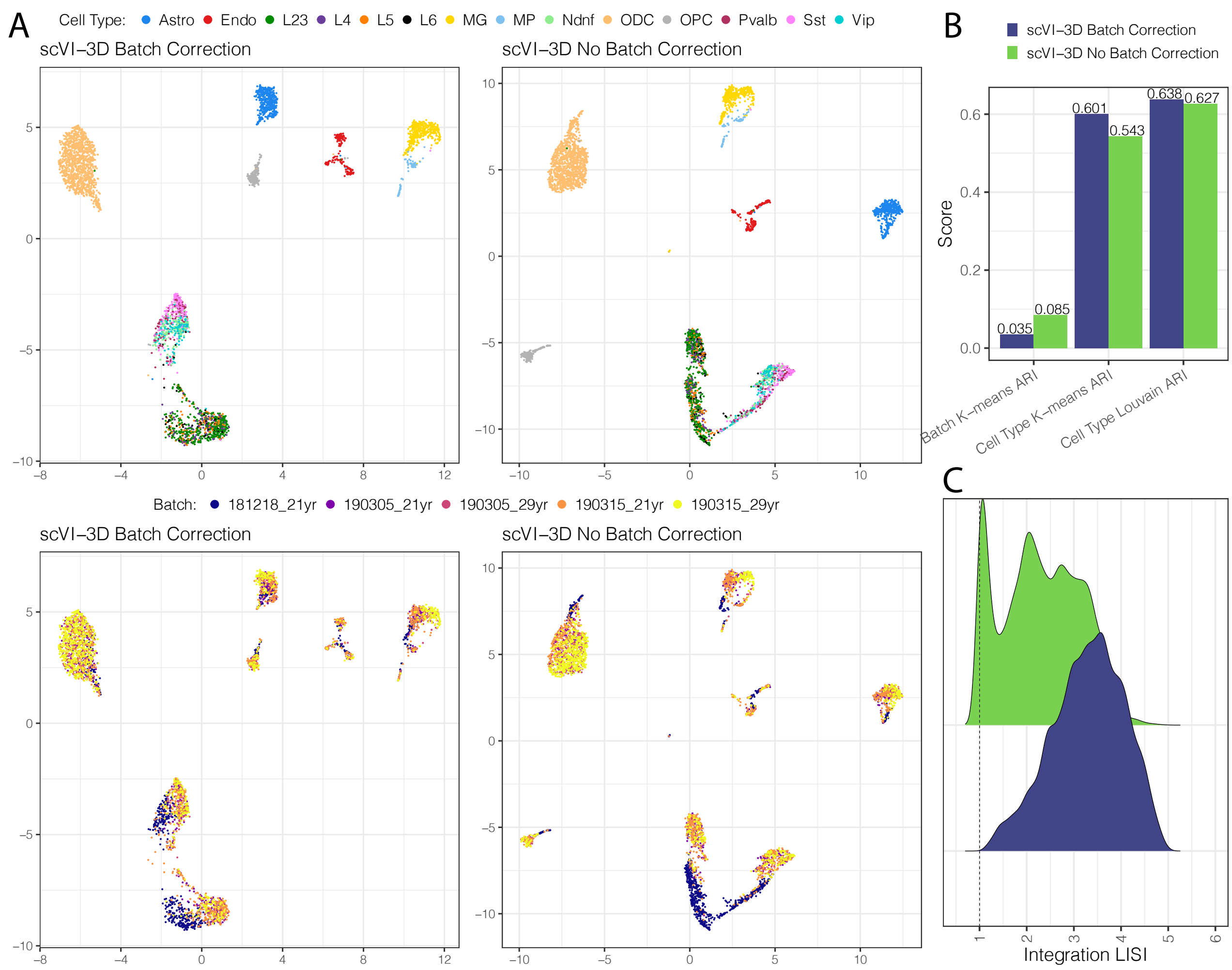

Fig. S15 *Batch correction improves the scVI-3D normalization performance on* `Lee2019` *data set.* **A.** UMAP visualization comparing the cell type separation and batch effect between scVI-3D with batch correction and without batch correction. **B.** ARI evaluation on the cell type separation and batch effect. **C.** Integration LISI scores distribution across cells that scVI-3D normalized with or without batch correction. A high density around the dashed line ($x = 1$) indicates only one batch in a particular cell's neighborhood, demonstrating the batch effect. Larger values indicate well mixing across the batches.

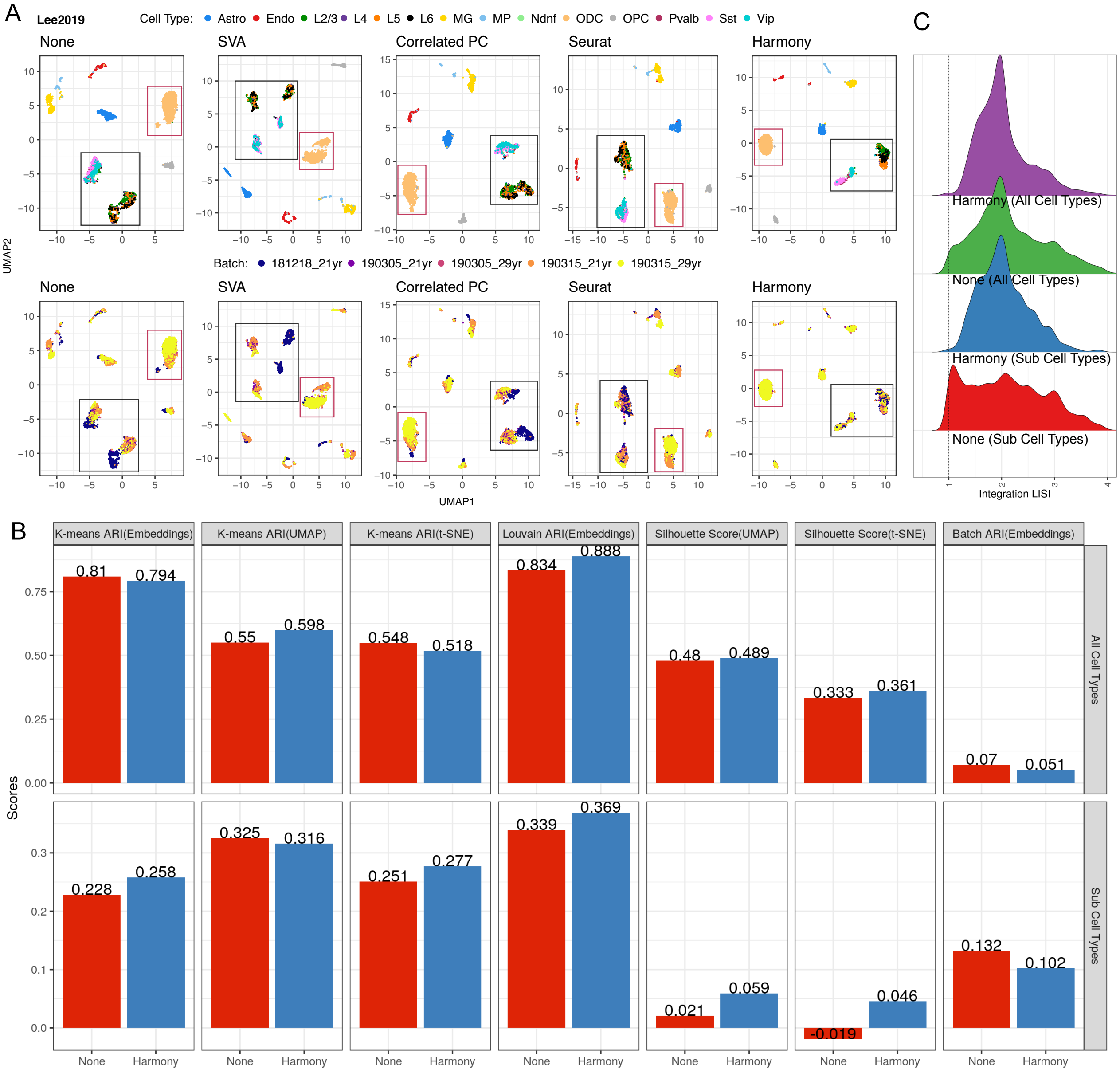

Fig. S16 *Performance comparison of the batch effect correction methods coupled with BandNorm on* `Lee2019` *data set.* **A.** Results from the four batch effect removal methods, SVA [33], removing the top correlated principal component, Seurat batch effect regression [34], and Harmony [25], together with the BandNorm normalization on the `Lee2019` data set. The ODC cell type is highlighted in the red square, and excitatory and inhibitory neuronal subtypes are highlighted in the black square for direct comparison across batch removal methods. **B.** ARI and Silhouette scores evaluations on the cell type separation and batch effect comparing the BandNorm normalization with and without Harmony batch correction. The evaluations are done on all the cell types in first row panels and on excitatory and inhibitory cells (L2/3, L4, L5, L6, Ndnf, Pvalb, Sst, and Vip) in the second-row panels. **C.** The integration local inverse Simpson'sSimpson's Index (iLISI) assessment [25] of the batch effect using BandNorm with or without Harmony correction on all the cell types or sub-neural excitatory and inhibitory cell types that were more severely affected by the batch effect. A high density around the dashed line ($x = 1$) indicates only one batch in a particular cell's neighborhood, demonstrating the batch effect. Larger values indicate well mixing across the batches.

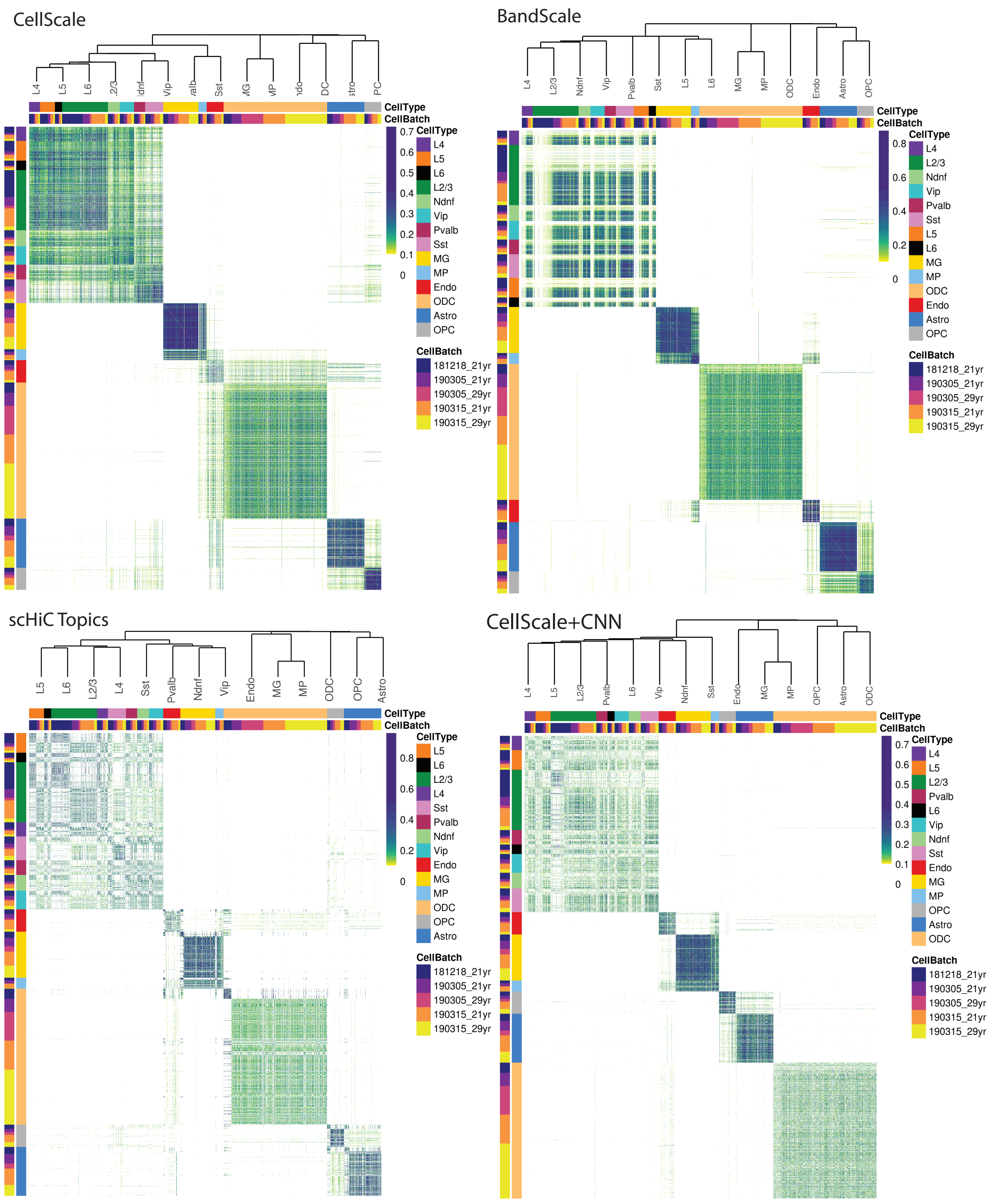

Fig. S17 *Pairwise cell similarities based on low-dimensional embeddings to elucidate cell type relationships.* Pairwise cell similarity scores for the Lee2019 data set are obtained by edge weights of the shared nearest neighbor graphs constructed from normalized data (low dimensional embeddings from scHiC Topics, CellScale+CNN, and first 50 PCs for CellScale and BandScale). The dendrogram depicts the inferred relationships between the cell types from the hierarchical clustering of the cells.

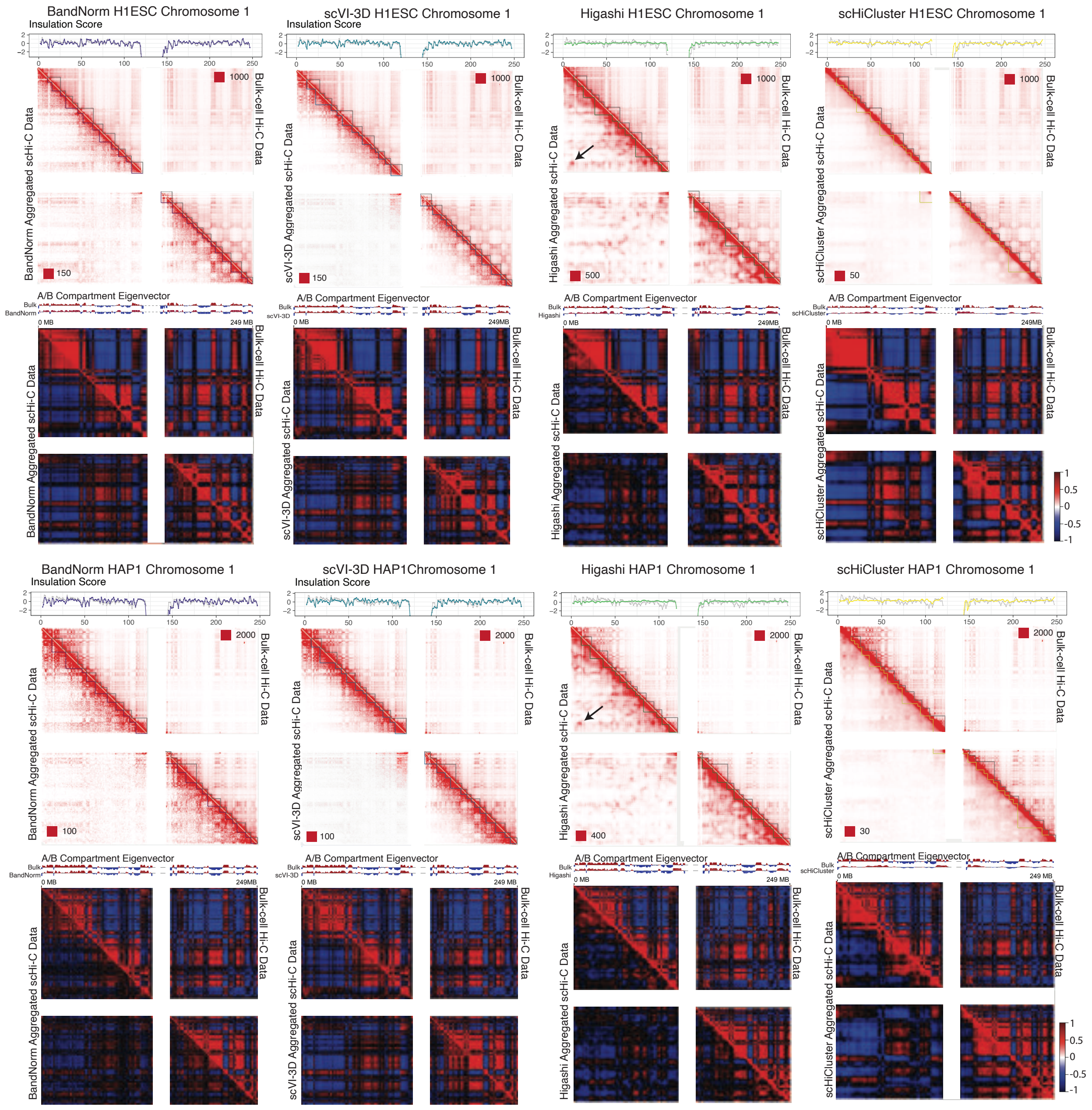

Fig. S18 *Evaluation for the detection of topologically associating domains (TADs) and A/B compartments in H1ESC and HAP1 cells.* Comparison of detected TADs and A/B compartment between bulk Hi-C data (upper right triangles) and the aggregated single-cell Hi-C data (lower left triangles) after normalization or de-noising on Kim2020 data set with known H1ESC and HAP1 cell type labels. The numbers after the red squares at the left bottom or right upper corner of each contact matrix represent the minimum interaction frequency for the reddest locus-pair. The black arrows highlight one example region that keeps showing over-imputation artifacts by Higashi across all five cell types compared to the bulk Hi-C data as a gold standard. The insulation scores [80] that trace the TAD boundaries are depicted above the contact matrices with grey lines corresponding to bulk Hi-C data and purple for BandNorm, blue for scVI-3D, green for Higashi, and yellow for scHiCluster. A/B compartments are detected using the eigenvector of the correlation map of bulk (upper right triangles) or aggregated (lower left triangles) Hi-C matrices, values of which are displayed above each correlation matrix.

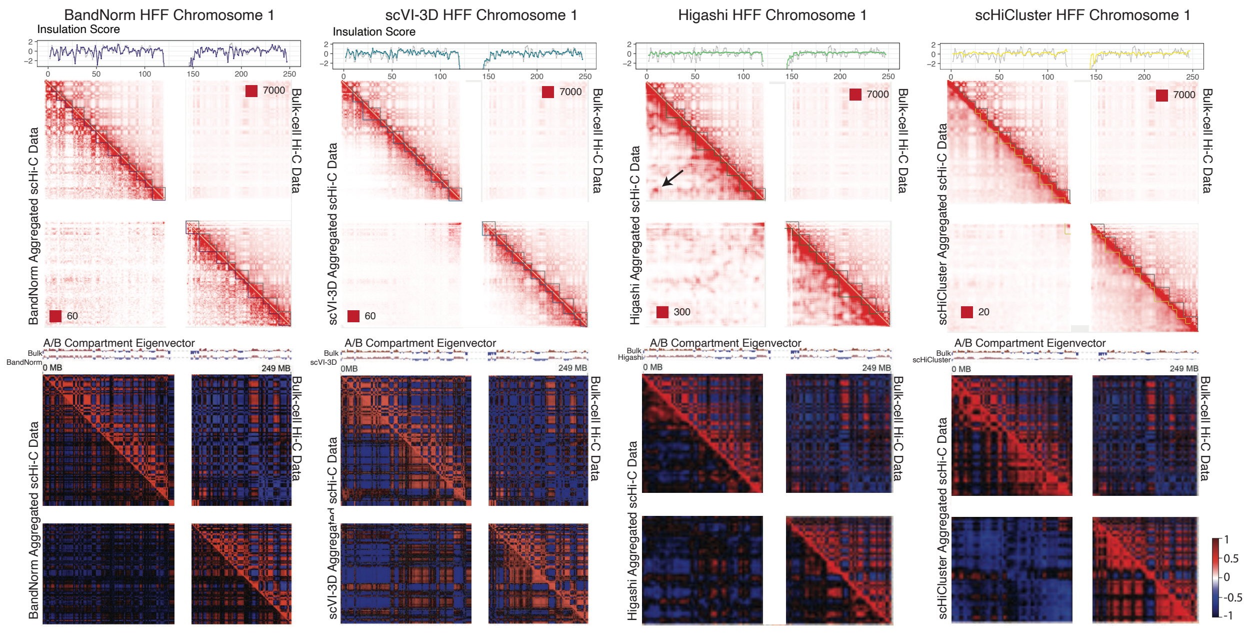

Fig. S19 *Evaluation for the detection of topologically associating domains (TADs) and A/B compartments in HFF cells.* Comparison of detected TADs and A/B compartment between bulk Hi-C data (upper right triangles) and the aggregated single-cell Hi-C data (lower left triangles) after normalization or de-noising on `Kim2020` data set with known HFF cell type labels. The numbers after the red squares at the left bottom or right upper corner of each contact matrix represent the minimum interaction frequency for the reddest locus-pair. The black arrow highlights one example region that keeps showing over-imputation artifacts by Higashi across all five cell types compared to the bulk Hi-C data as a gold standard. The insulation scores [80] that trace the TAD boundaries are depicted above the contact matrices with grey lines corresponding to bulk Hi-C data and purple for BandNorm, blue for scVI-3D, green for Higashi, and yellow for scHiCluster. A/B compartments are detected using the eigenvector of correlation map of bulk (upper right triangles) or aggregated (lower left triangles) Hi-C matrices, values of which are displayed above each correlation matrix.

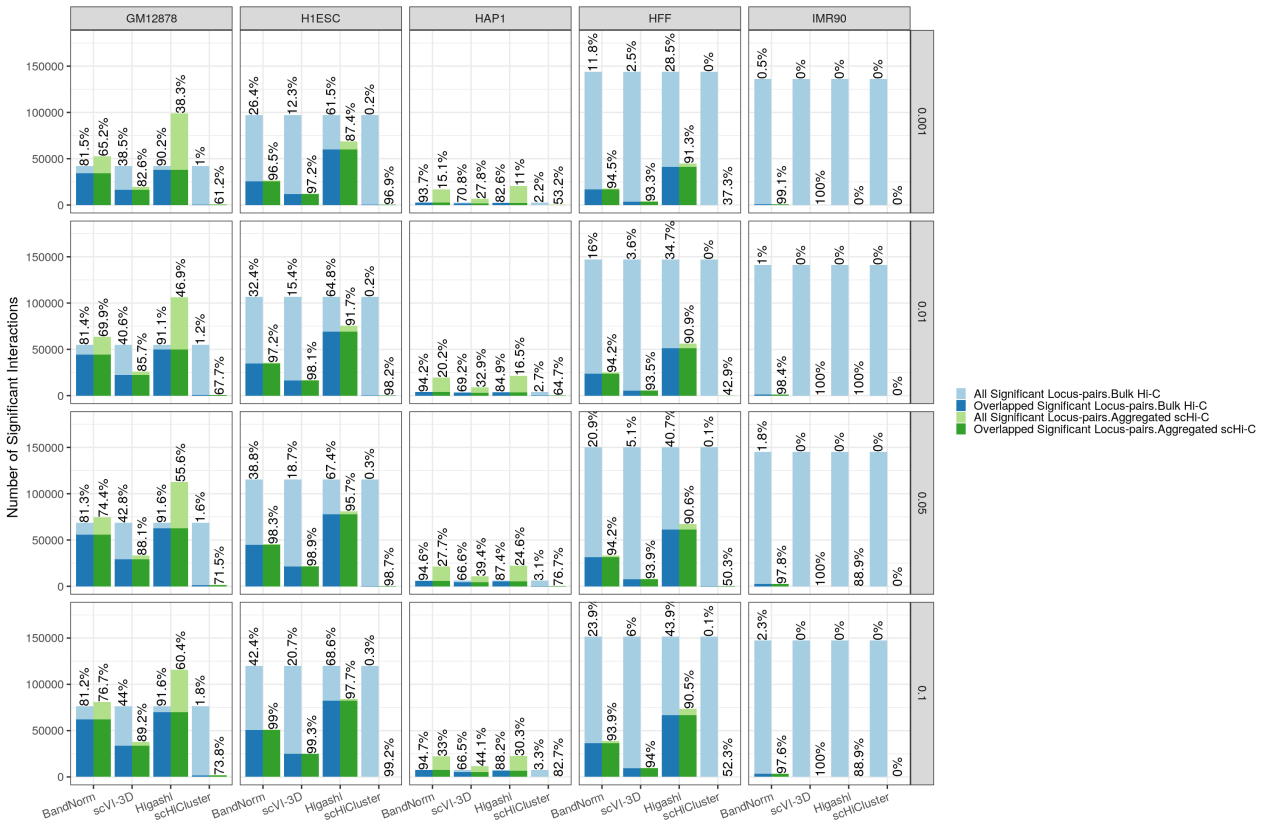

Fig. S20 *The number of significant locus-pairs called by Fit-Hi-C on bulk or aggregated data*. Row panels correspond to different FDR thresholds (FDR $\leq$ 0.001, 0.01, 0.05, 0.1) to deem significant interaction locus-pairs. The percentage above each bar is the percentage of significant interaction detected in both bulk and aggregated Hi-C data.

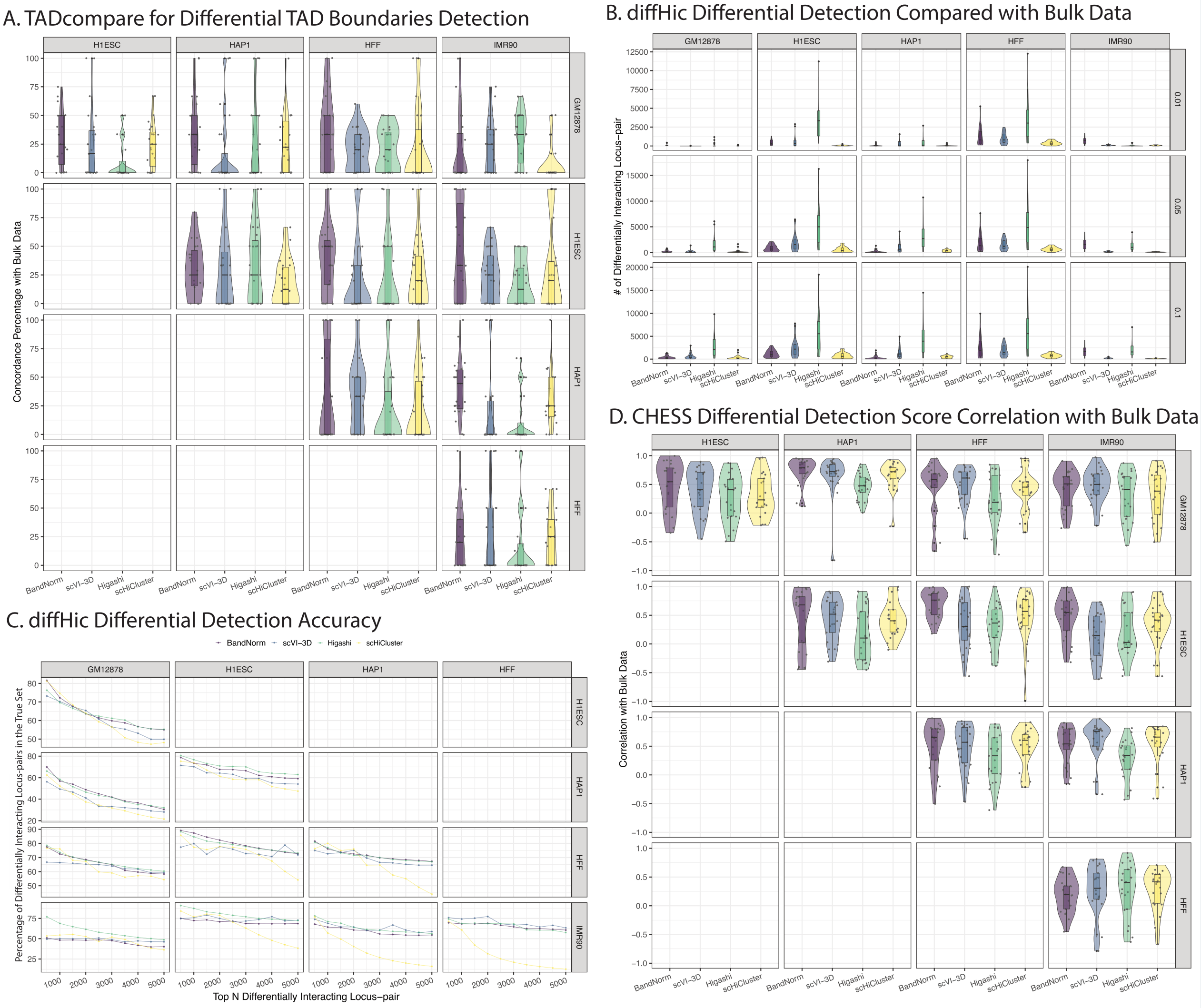

Fig. S21 *Domain and locus-pair differential analysis of aggregated scHi-C data after normalization and de-noising, with known true cell type labels.* **A.** Comparison of differential TAD boundaries detected by TADcompare [38] between every pair of cell types. **B.** Comparison of differentially interacting locus-pairs detected between bulk and aggregated scHi-C after normalization or de-noising, using diffHic [39]. The three rows correspond to filtering of the differential chromatin interactions at three FDR levels, i.e., adjusted P value ≤ 0.01 for the first row. **C.** Percentage of top N (N = 500, 1,000, ...) significant differentially interacting locus-pairs, detected by diffHic [39] analysis of aggregated scHi-C matrices from each method, that are in the gold standard set. The gold standard set is defined as the significant differentially interacting locus-pairs detected by diffHic [39] from the cell type specific bulk Hi-C data. **D.** Correlation of CHESS [40] scores, depicting differential interactions of the cell types, between bulk Hi-C and aggregated scHi-C from different methods. Sample sizes for each violin plot of **A, B, D** are n = 23 corresponding to 23 chromosomes investigated.

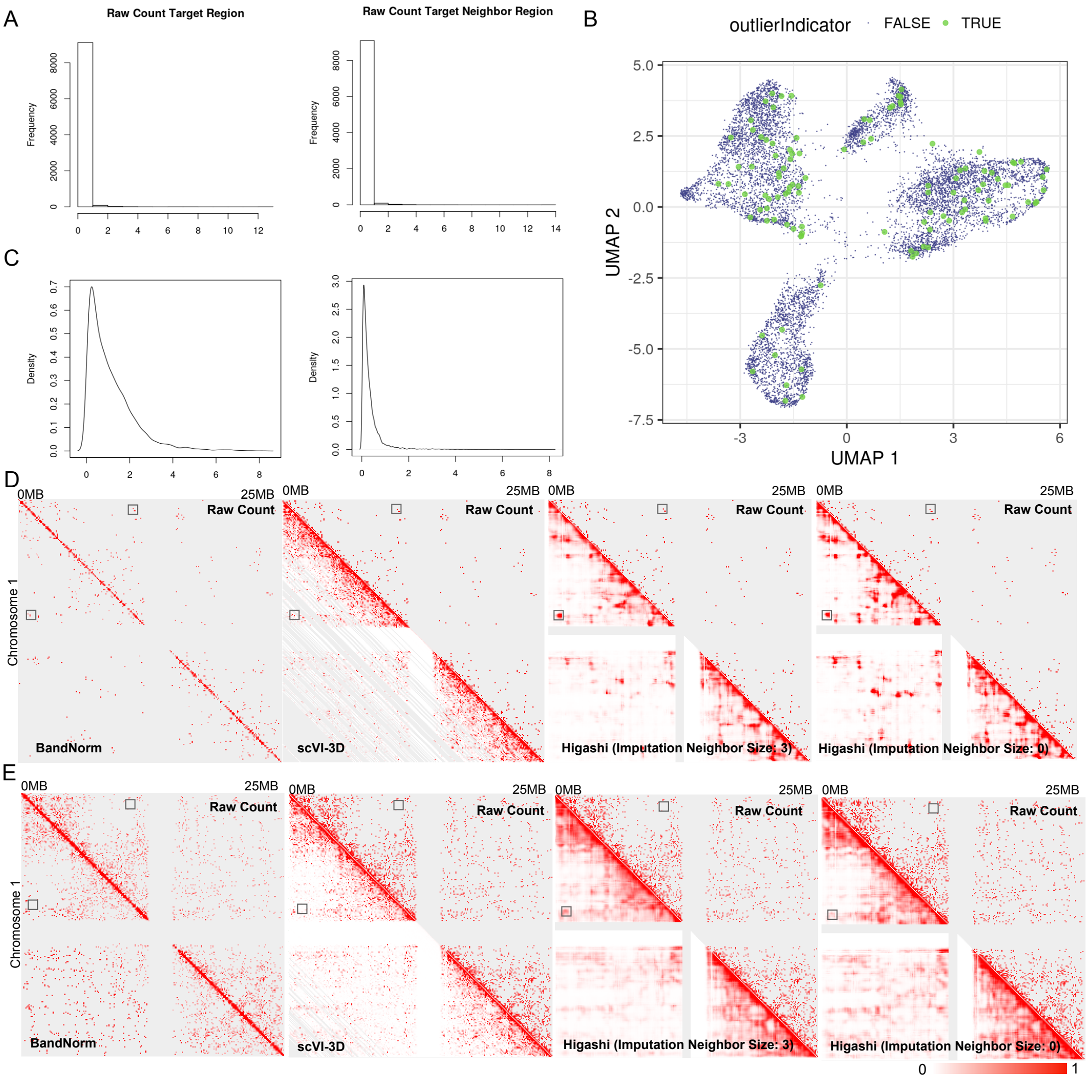

Fig. S22 *Over-enrichment artifacts in off-diagonal regions by Higashi imputation*. One example of the over-enriched genomic regions in the Higashi imputed contact maps were identified as the interaction between chr1:7-12Mb and chr1:109-114Mb (highlighted by arrows in Figs. 5-6 and Additional file 1: Figs. S18-19 as the target region). Its immediate neighborhood of chr1:13-18Mb interacting with chr1:109-114Mb is utilized as a control for comparison in our investigation. **A.** Histogram of the raw contact counts across individual cells in the target and control neighborhood regions. Both regions share a similar distribution, with most cells having zero or only 1 interaction. **B.** The cells that have more than 1 interaction in the target region are labeled. Such outlier cells are distributed across all the cell-type clusters. **C.** Density of the Higashi imputed interaction counts at the target and control regions. The target region has markedly more cells with relatively higher counts than the neighborhood control region. This is consistent with the visualization of target region enrichment in Figs. 5-6 and Additional file 1: Figs. S18-19. **D.** Contact matrix visualization for one example outlier cell of GM12878 that has 4 raw interactions in the target region. Comparison is across contact maps of the raw counts and counts normalized or de-noised by BandNorm, scVI-3D, and Higashi. The target region is marked with grey squares. The original artifact is significantly enlarged in the Higashi-generated contact matrix. “neighbor_num” is set to 3 or 0 instead of the default value 5 to include fewer neighbor cells or no neighbor cells in the imputation process. **E.** Contact matrix visualization for another example cell of GM12878 that has zero contact count in the target region. Outlier cells' signal artifacts tend to affect neighboring cells, leading to over-enrichment in the target region. With the “neighbor_num” parameter of Higashi software being 0, namely, no information from the neighbor cell is borrowed, the over-imputation is attenuated but not completely eliminated.

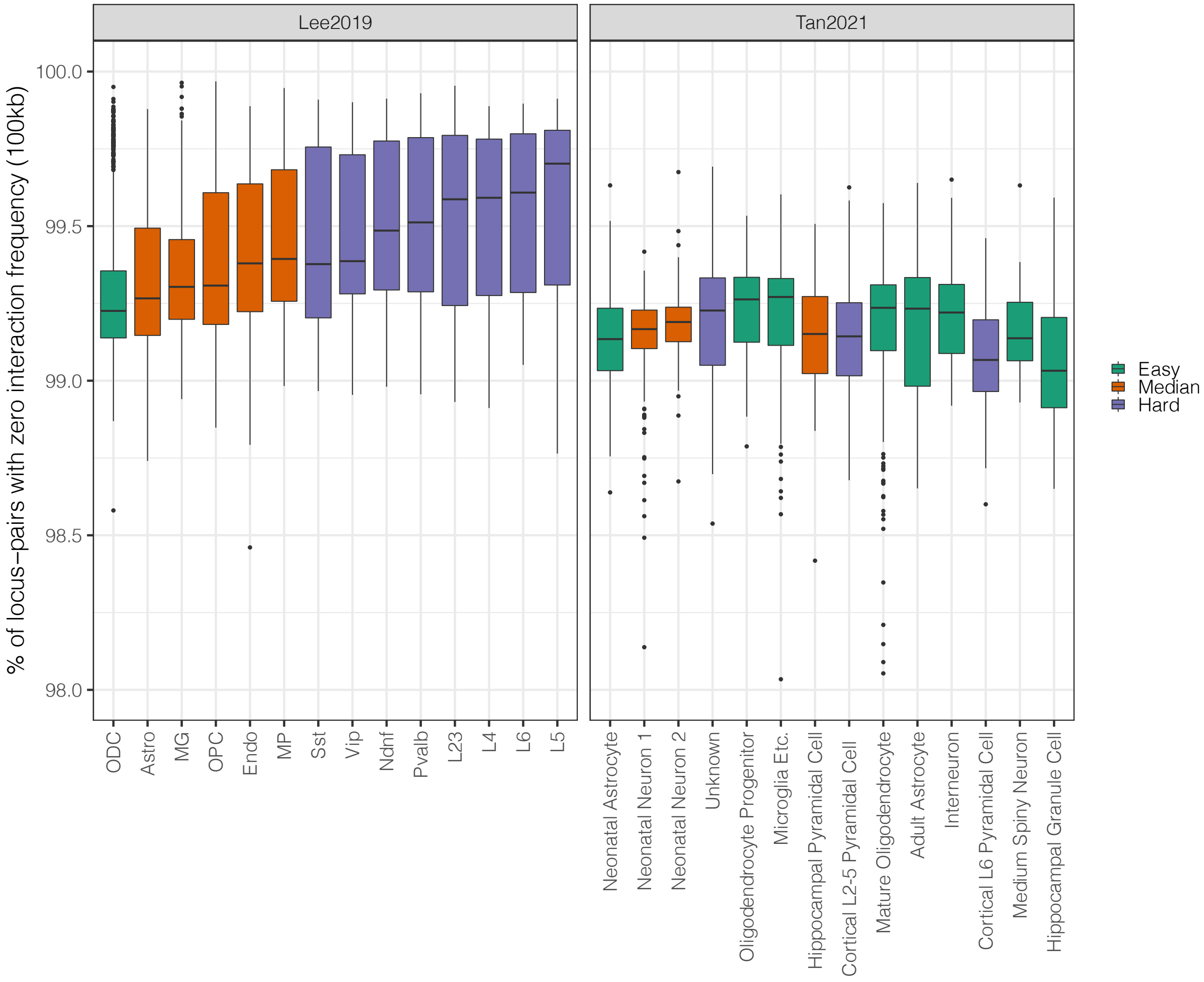

Fig. S23 *Comparison of sparsity levels between `Lee2019` and `Tan2021` data sets at 100kb resolution.* The percentage of locus-pairs with zero interaction frequency per cell (i.e., sparsity) for `Lee2019` and `Tan2021` data set at 100kb resolution is grouped by cell type. Cell types are colored by the how difficult the cell type is distinguishable from other cell types based on UMAP visualizations.

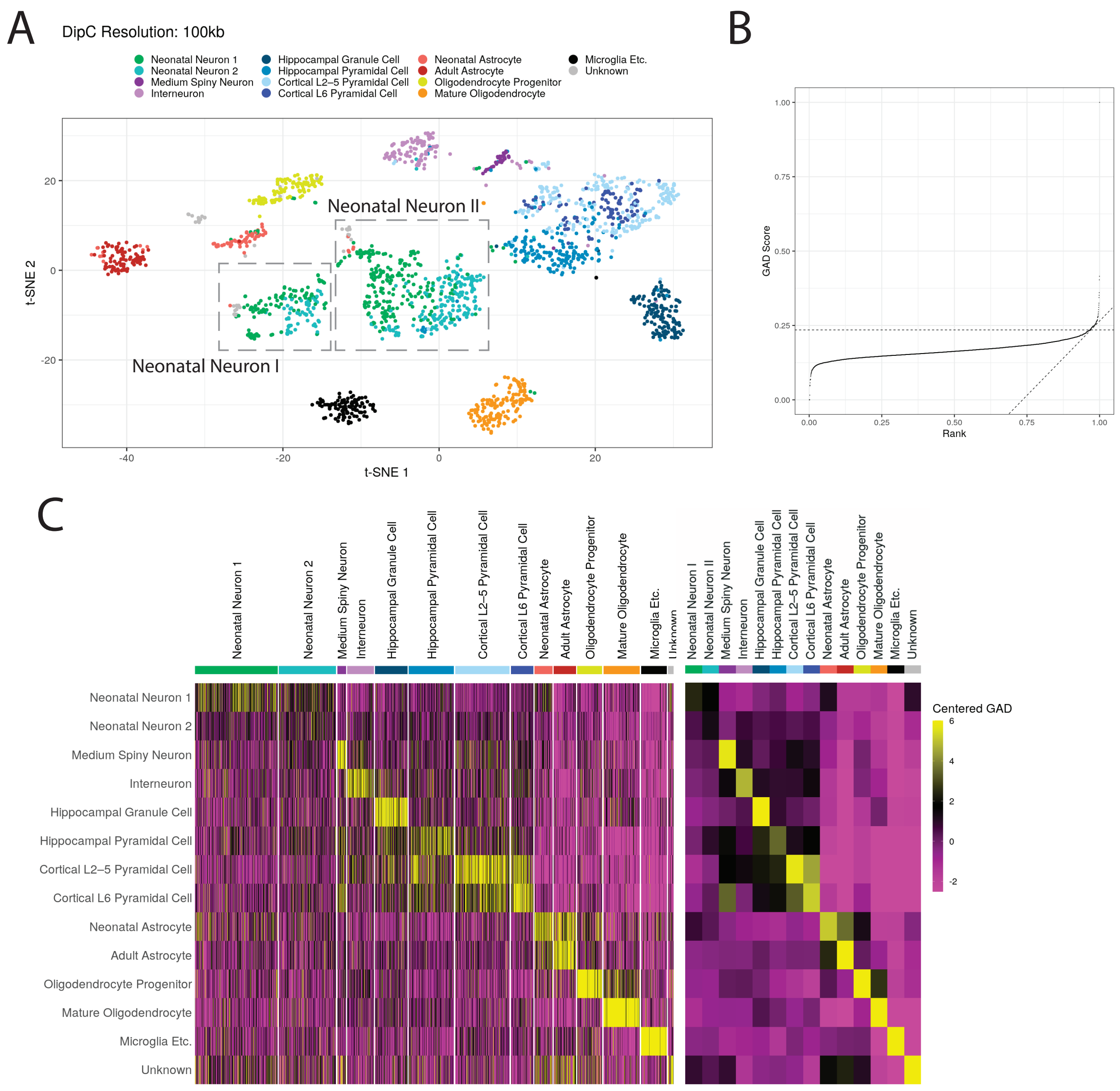

Fig. S24 *scGAD score analysis of* Tan2021 *data set.* **A.** t-SNE project of single-cell Dip-C data of Tan2021 data set [11]. **B.** Ranked average scGAD scores for genes longer than 100kb. Genes are ordered by the average scGAD scores across all the cells on the x-axis. Both the rank and scGAD scores are scaled to unit interval $[0, 1]$. The threshold to identify significant GAD formations is defined as in Tan et al. 2021 [11] and captures genes above the tangent of the curve. **C.** Analogue of Fig. 9d using the original neonatal neuronal sub-cluster labels from Tan et al. 2021 [11] (Neonatal Neuron 1 and Neonatal Neuron 2). scGAD scores of lists of cell-type-specific marker genes, detected by comparing scGAD scores across cell types, among single cells (left panel) and averaged for each cell type (right panel). scGAD score values are standardized into z-scores.

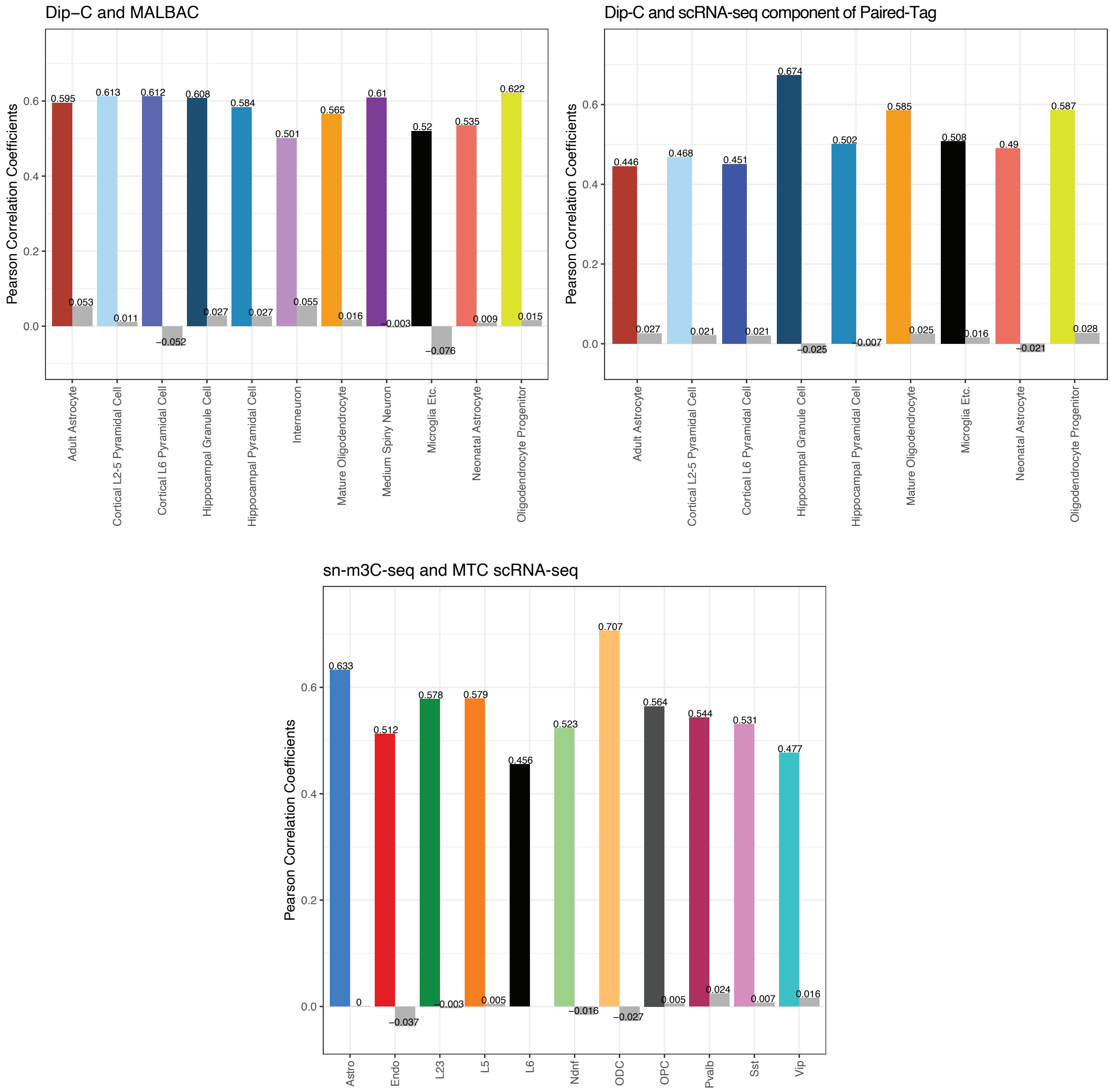

Fig. S25 *Pearson correlation coefficients between scGAD and scRNA-seq.* Pearson correlation coefficients measure the correlation between scGAD scores and scRNA-seq readouts of cells from the same cell type system. scGAD scores and scRNA-seq readouts are average across cells of the same cell type, respectively, and the correlation is calculated across eligible genes (i.e., 100kb or longer genes and have gene expression and interaction frequency). The grey bars depict the correlation baseline where the cells are randomly permuted across cell types. Dip-C and MALBAC are from Tan et al. 2021 [11]. Paired-Tag data set is from Zhu et al. 2021 [81]. sn-m3C-seq measures methylation and 3D chromatin organization simultaneously in each cell, and the data is from Lee et al. 2019 [10]. The corresponding scRNA-seq data for Lee2019 is from Bakken et al. 2021 [82].

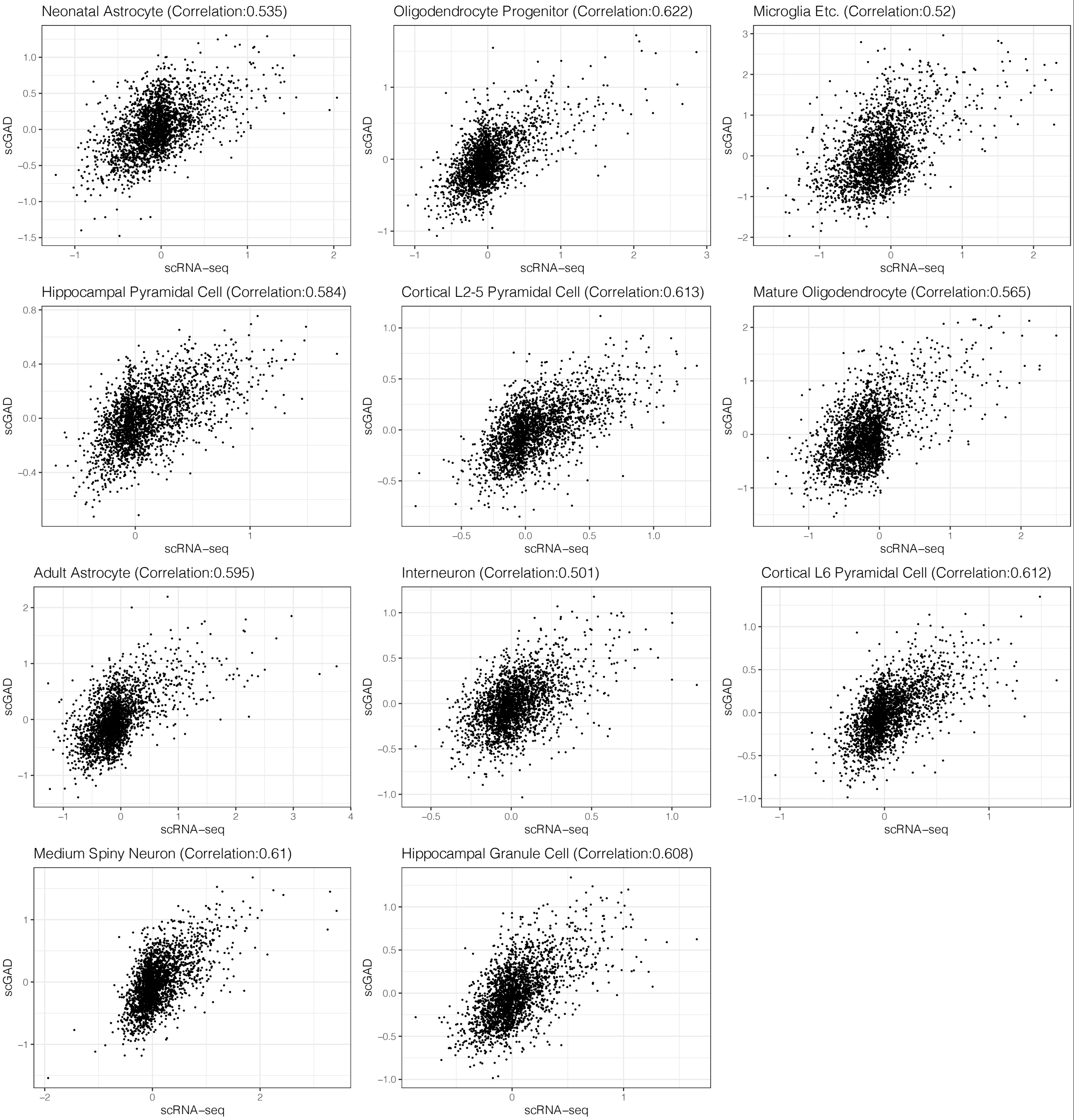

Fig. S26 *Relationship between scGAD scores of Dip-C measurement and scRNA-seq readouts of MALBAC profiling for each cell type from Tan2021 data set.*

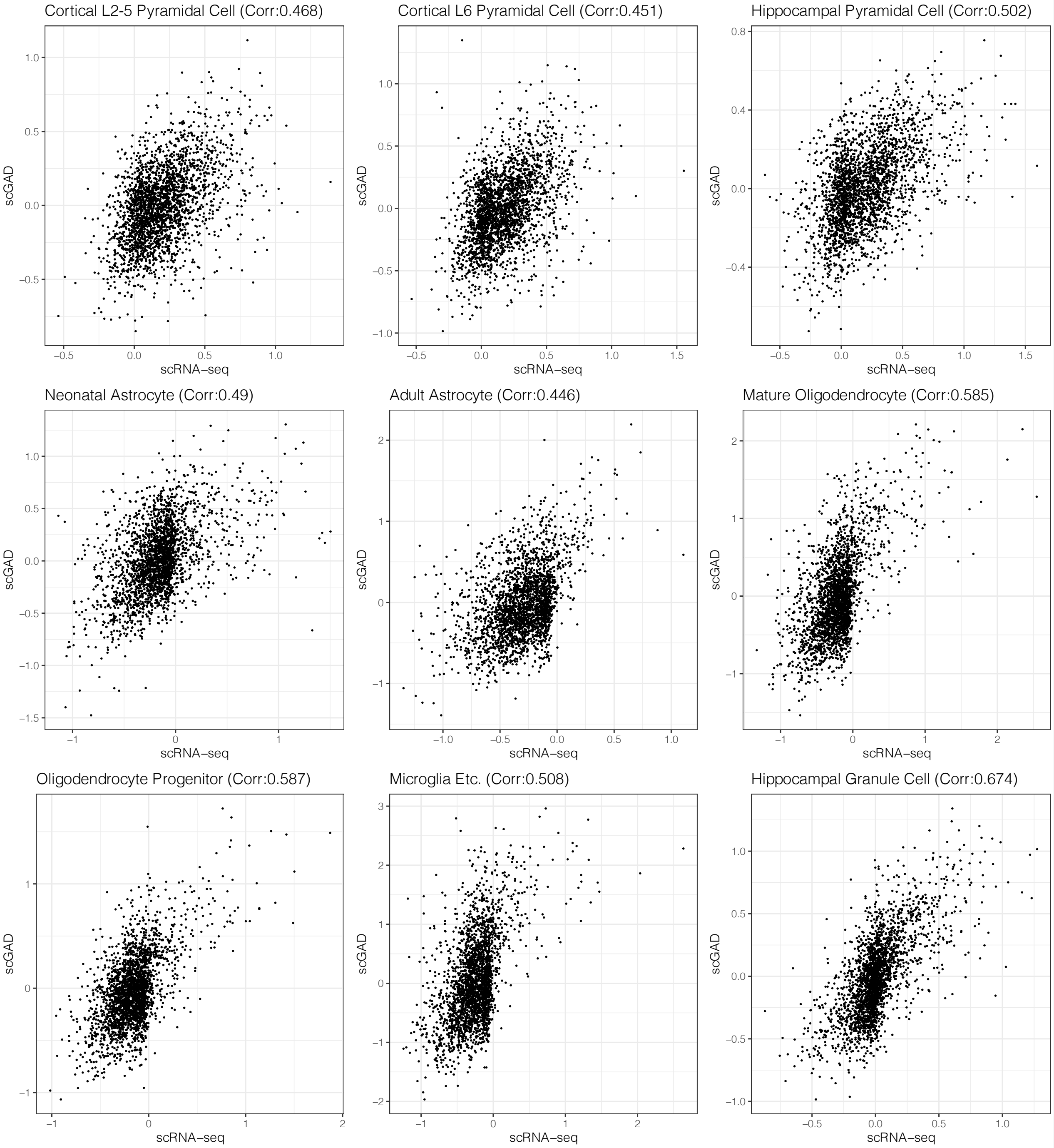

Fig. S27 *Relationship between scGAD scores of Dip-C measurement from* `Tan2021` *data set and scRNA-seq component of Paired-Tag data [81].*

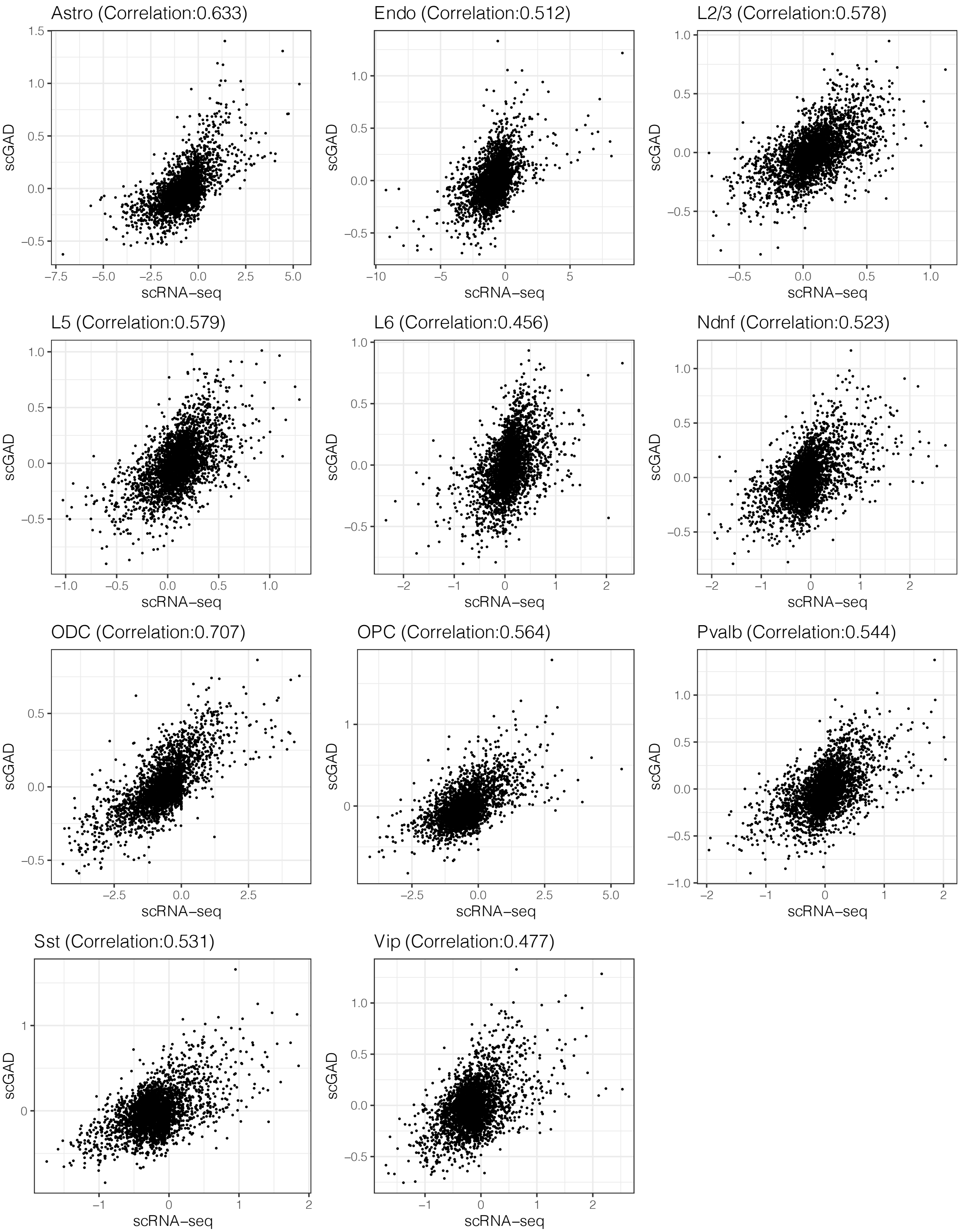

Fig. S28 *Relationship between scGAD scores of sn-m3C-seq measurement from* `Lee2019` *and scRNA-seq readouts from the BRAIN Initiative [82].*

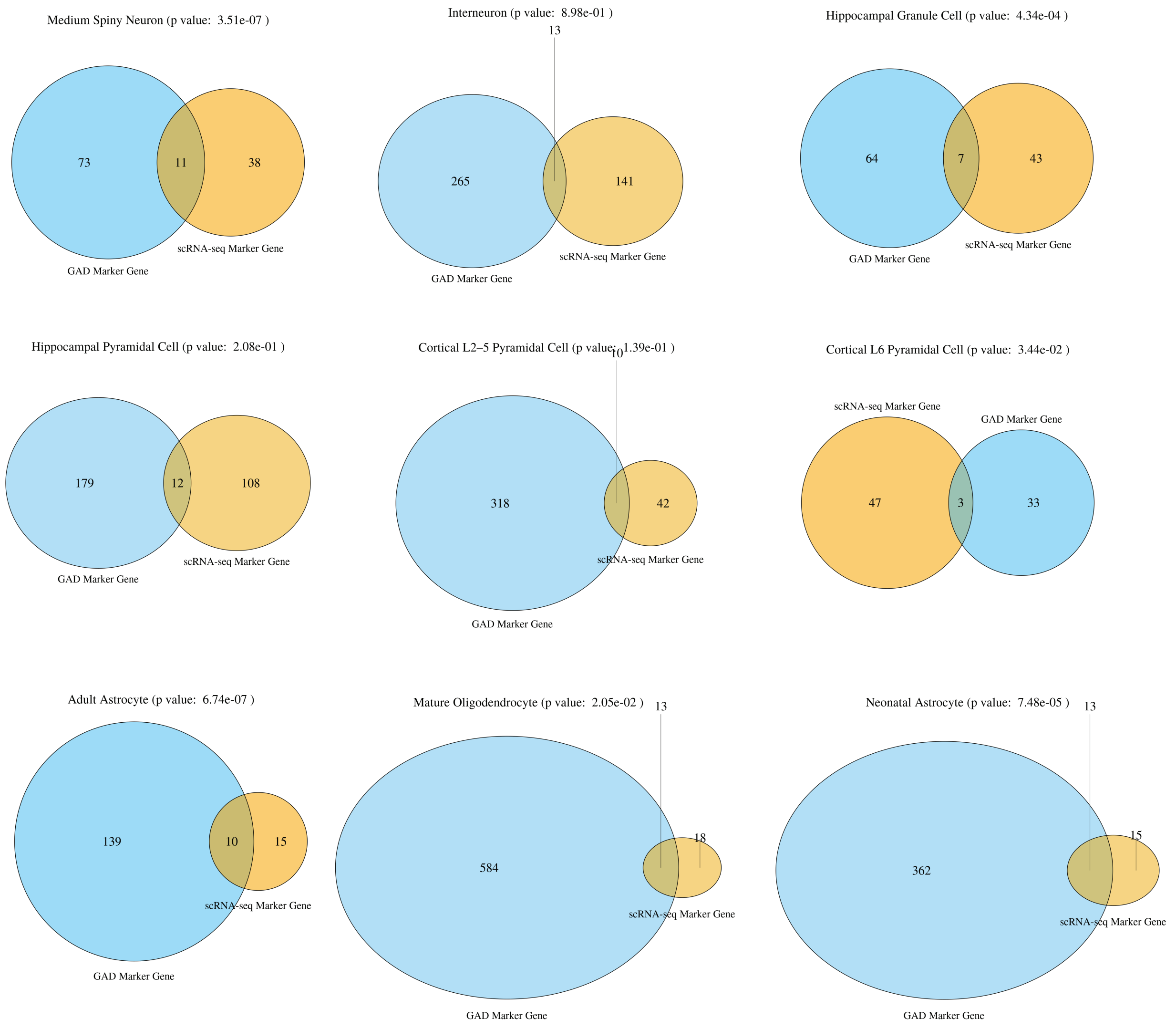

Fig. S29 *Overlap between scGAD marker genes and marker genes identified from single-cell transcriptomics data.* Blue circles represent the number of marker genes detected by comparing scGAD scores across cell types. Yellow circles represent those detected by MALBAC-DT. The significance of the overlap is evaluated by the Fisher's exact test.

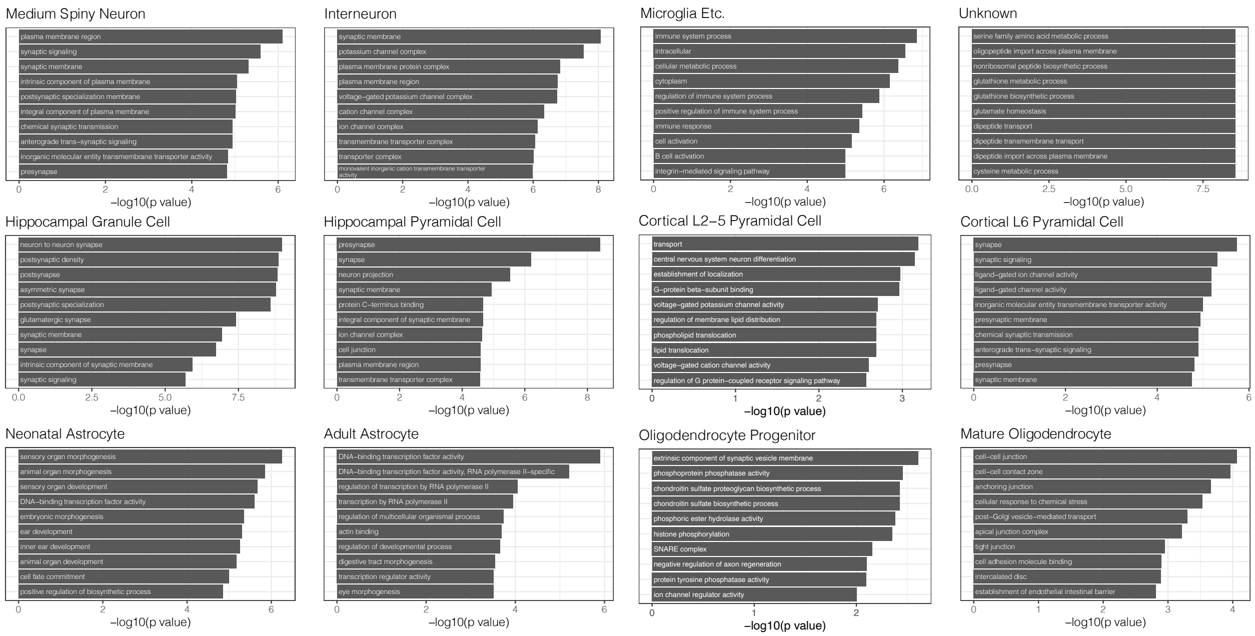

Fig. S30 *Gene ontology analysis of cell-type specific marker genes defined by scGAD scores.* The cell-type specific marker genes are detected by "FindMarkers" function of Seurat R package based on the scGAD scores on individual cells. Gene ontology analysis is performed with the "goseq" R package.

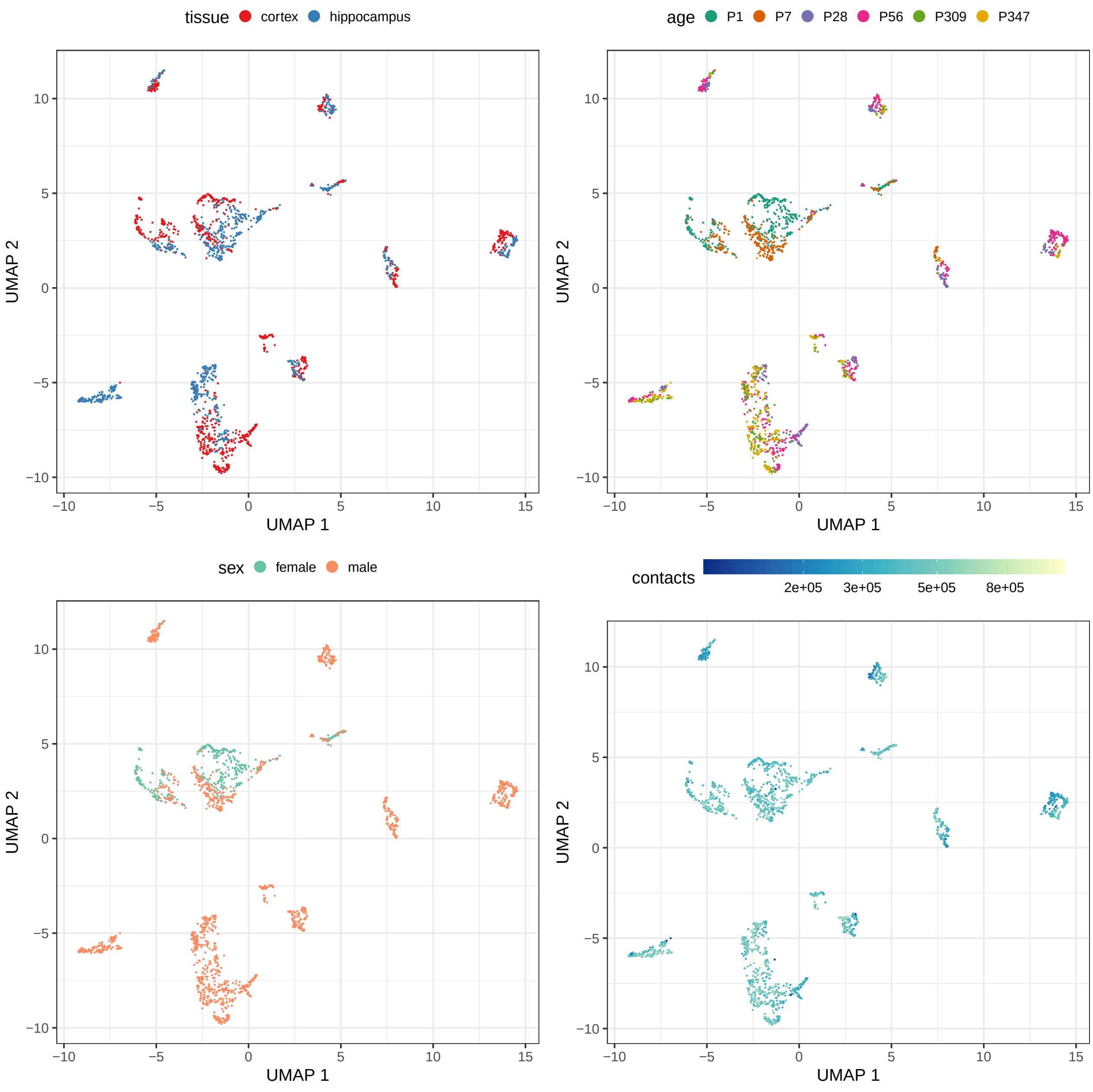

Fig. S31 *Tissue, age, sex and contact counts of single-cell Dip-C data.* The distribution of tissue, age, sex, and contact counts of individual cells on the UMAP projection of the Dip-C data from `Tan2021` [11].

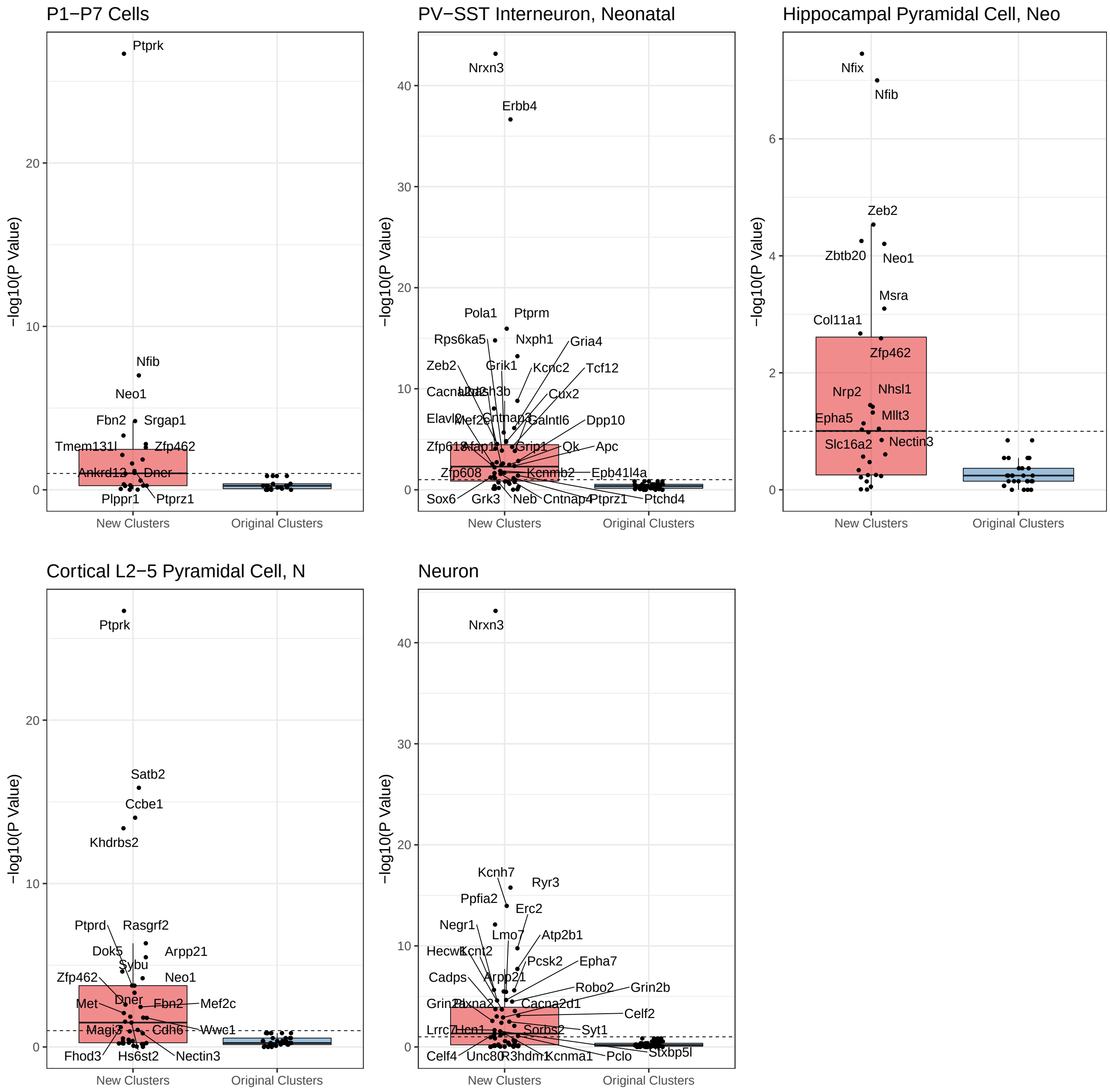

Fig. S32 *Transformed p values from the comparison of scGAD scores of marker genes across the two neonatal neuronal sub-clusters.* "New clusters" and "Original clusters" depict the sub-cluster labels obtained in this paper and Tan et al. 2021 [11], respectively. The marker genes are inferred by Tan et al. 2021 [11] from the neonatal single-cell transcriptomics harboring PV/SST Interneuron neonatal cells, Hippocampal Pyramidal neonatal cells, and Cortical L2-5 Pyramidal neonatal cells from postnatal day 1 and 7. P values are obtained by the Wilcoxon rank-sum test.

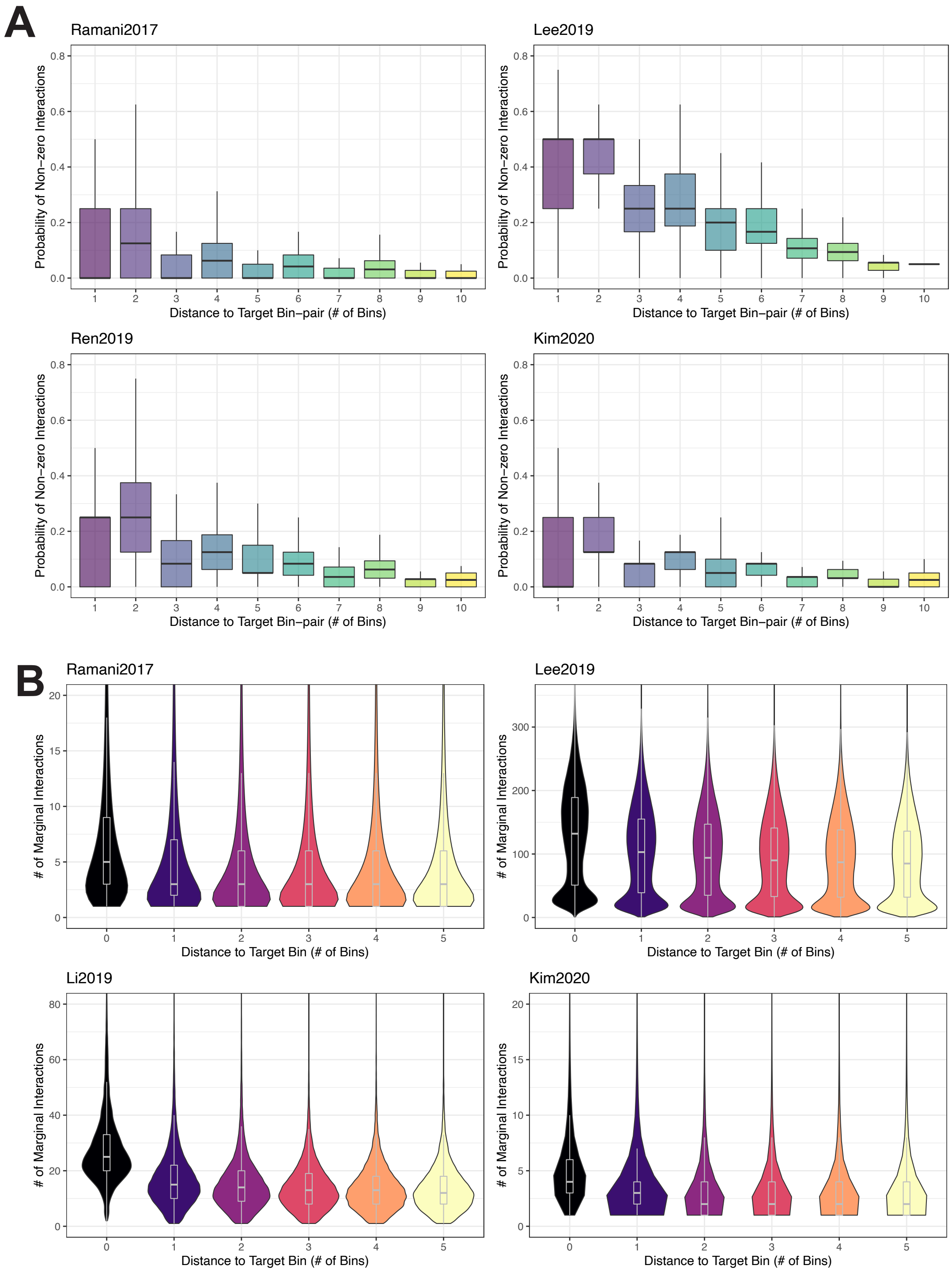

Fig. S33 *Neighborhood effect at 1Mb resolution.* **A.** Empirical probability of non-zero interaction around the neighboring locus-pairs of top 10 locus-pairs with interaction frequencies (referred to as high IF locus-pairs) ≥ 3 on each chromosome contact matrix per cell. Distance is defined as the number of locus-pairs between the neighboring locus-pair and the high IF locus-pair. **B.** The genomic loci are first ordered based on their marginal interactions (row sum of the contact matrices) and the top 10 loci for each cell are considered per chromosome per cell. Boxplots depict the interaction frequencies around these loci as a function of genomic distance measured in numbers of locus-pairs between the high IF locus-pairs and their neighbors.

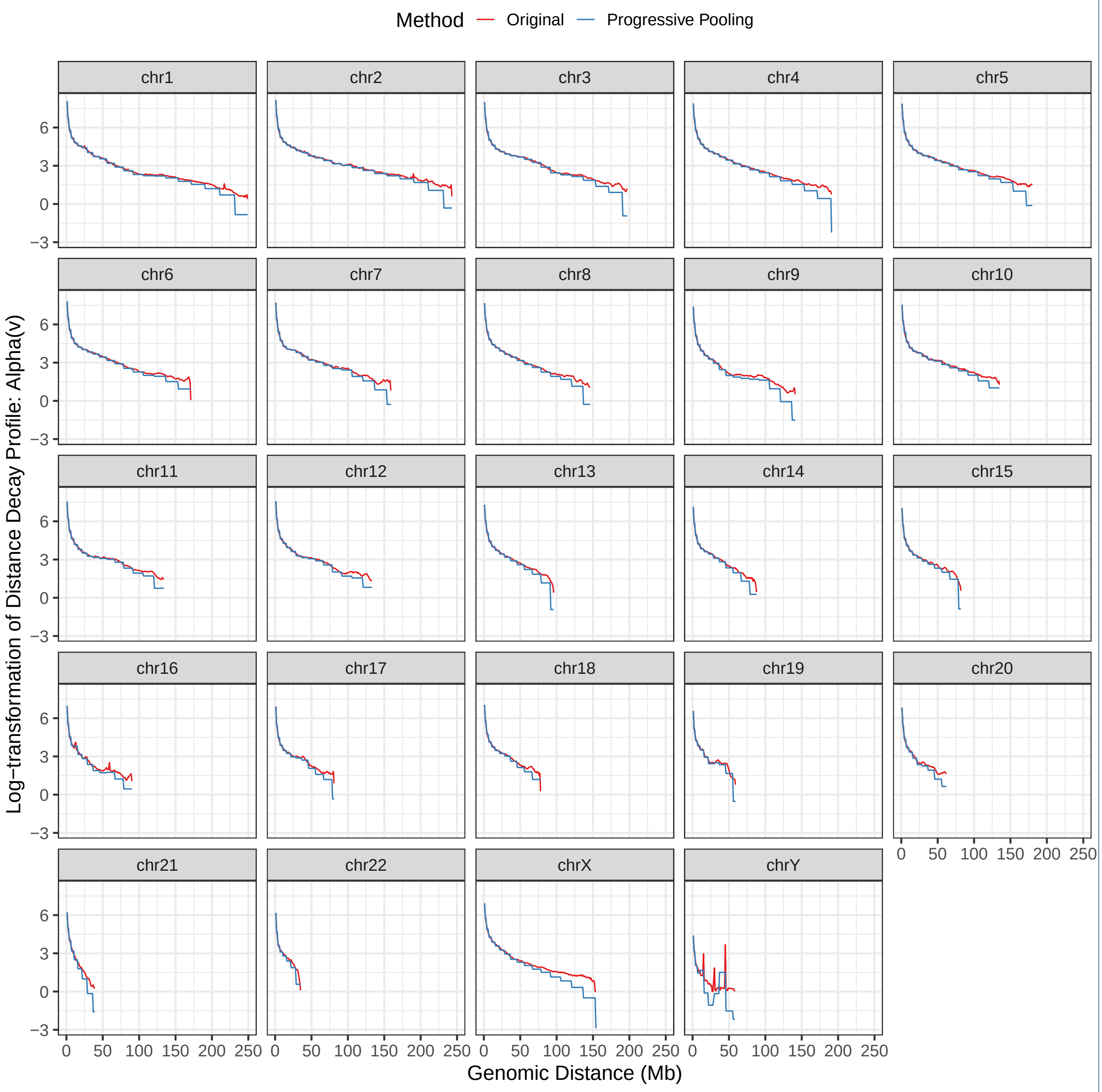

Fig. S34 *Distance decay profile change over genomic distance between raw count and counts after progressive pooling.*

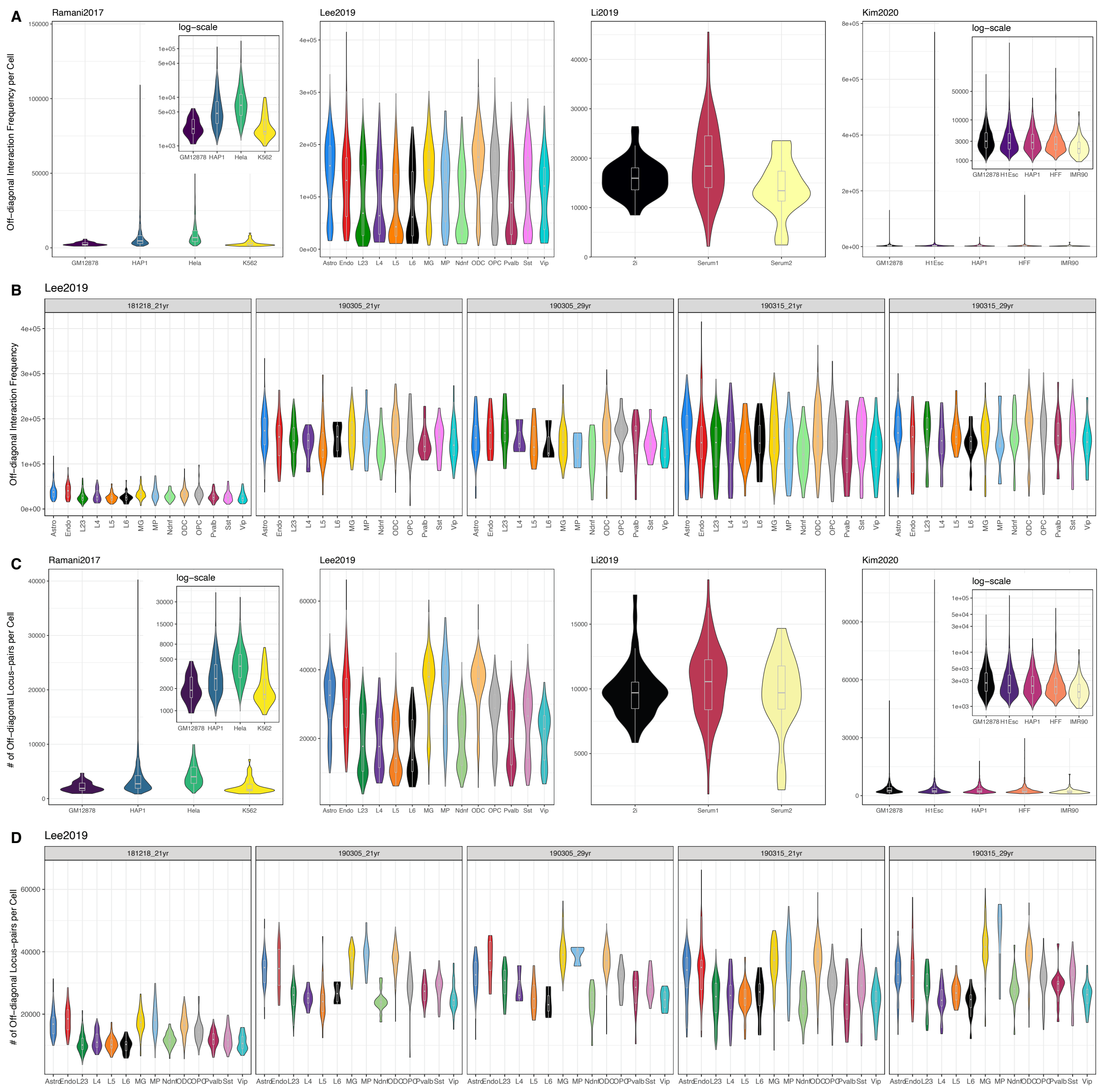

Fig. S35 *Distribution of the total off-diagonal interaction frequencies and the numbers of non-zero off-diagonal locus-pairs per cell.* **A.** Distribution of the total interaction frequencies across the contact matrices (excluding the main diagonal) of the cells for each cell type of `Ramani2017`, `Lee2019`, `Li2019`, and `Kim2020` data sets. The small panels within the figure display the same data with the y-axis is in log-scale. **B.** Total interaction frequencies per cell for the `Lee2019` data set stratified by the five libraries. The cells from the 181218_21 yr batch have relatively lower interaction frequency leading to a bimodal pattern in the cell-level total off-diagonal interaction frequencies in **A**. **C.** Summary of the total numbers of locus-pairs with non-zero interaction frequencies (excluding the locus-pairs on the main diagonal) per cell for each cell type of `Ramani2017`, `Lee2019`, `Li2019`, and `Kim2020` data sets. The small panels within the figure display the same data with the y-axis is in log-scale. **D.** Total number of non-zero off-diagonal locus-pairs per cell for the `Lee2019` data set stratified by the five libraries.

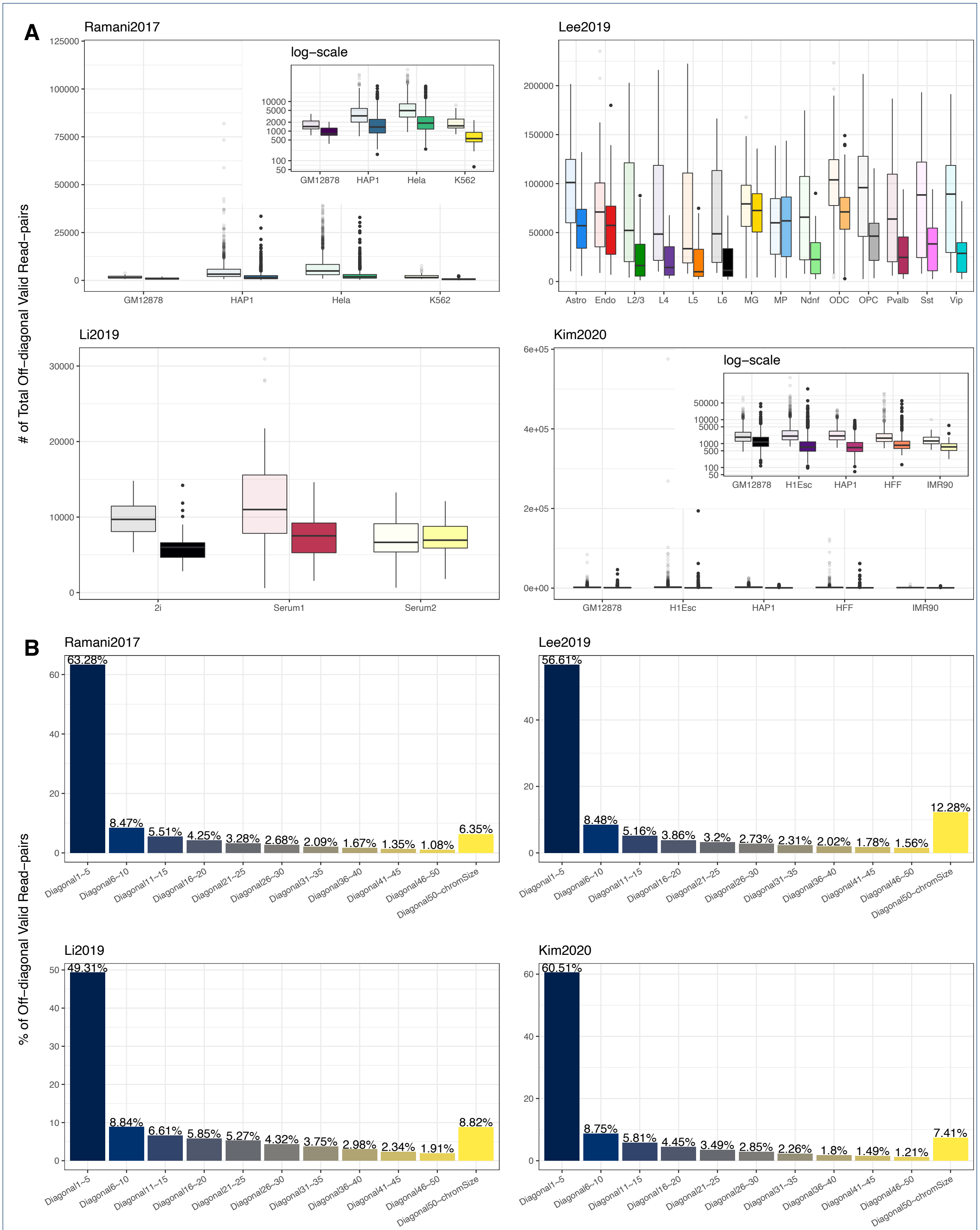

Fig. S36 *Genomic distance impact on the interaction frequency.* **A.** Total off-diagonal interaction frequency per cell for each cell type stratified by the interaction distance. First boxplot for each cell type depicts interactions between locus-pairs that are within 10Mb of each other and the second box plot per cell type depicts interactions between locus-pairs that are separated by more than 10Mb. **B.** Percentage of total interactions at different band intervals, excluding the matrix diagonal.

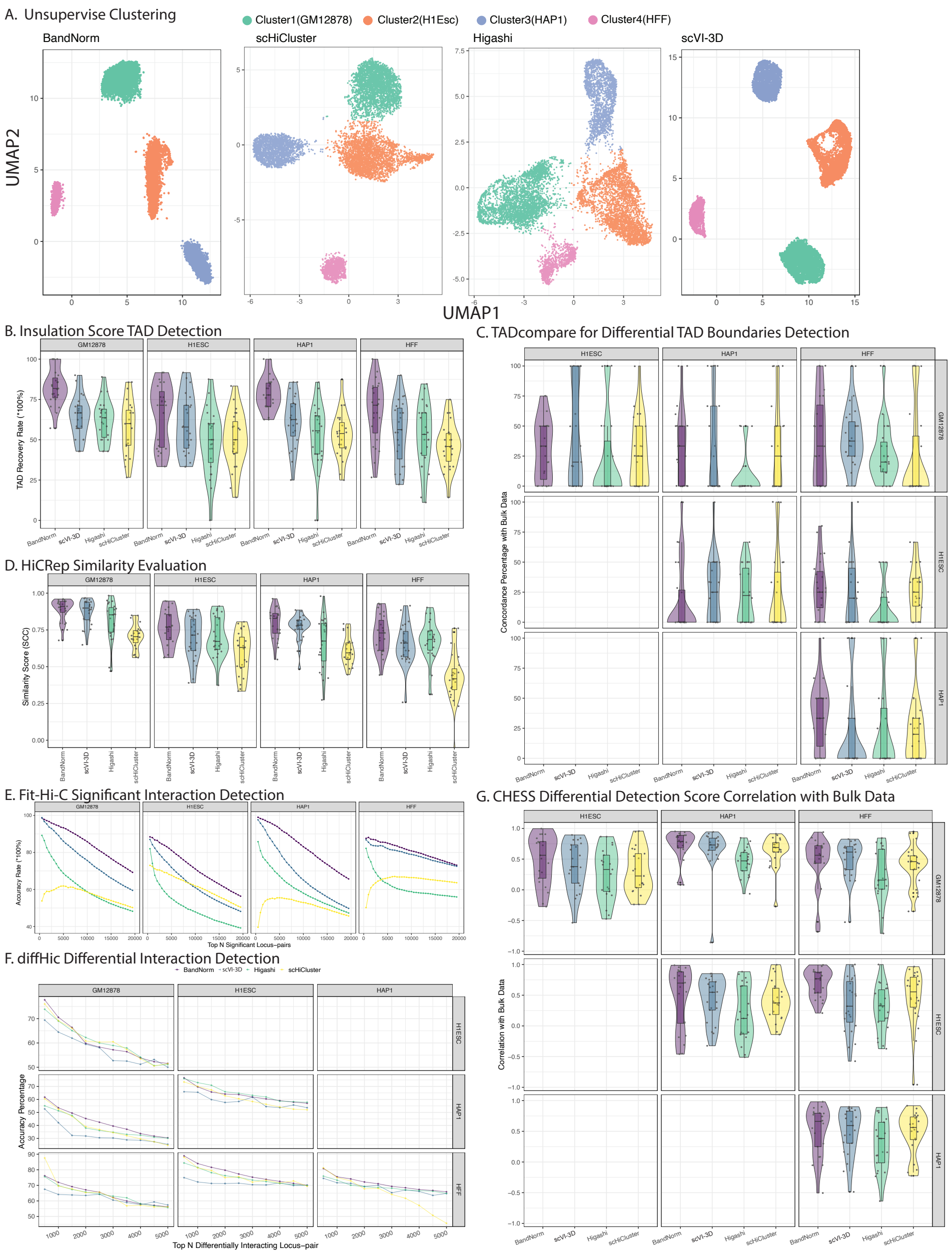

Fig. S37 *Evaluation of impact on downstream analysis of Hi-C data with cell type labels inferred from unsupervised clustering.* **A.** K-means clustering on the UMAP projections of low-dimensional latent embeddings of BandNorm, scHiCluster, Higashi, and scVI-3D. The clusters identified are labeled with cell types GM12878, H1ESC, HAP1, and HFF based on comparison with bulk Hi-C data. **B.** Percentage of TAD boundaries (based on Insulation Score [79]) that is within 1Mb distance of the corresponding bulk cell type Hi-C data TAD boundaries. **C.** Differential TAD boundaries detected by TADcompare [38] between every pair of clusters. **D.** HiCRep similarity of the aggregated scHi-C data of individual clusters with the bulk Hi-C data. **E.** Percentage of top N (N = 5,000, 10,000, ...) significant interacting locus-pairs that are in the gold standard set for each method. The gold standard set is defined as the top 50,000 significant locus-pairs detected by Fit-Hi-C [37] from the cell type specific bulk Hi-C data. **F.** Percentage of top N (N = 500, 1,000, ...) significant differentially interacting locus-pairs, detected by diffHic [39] analysis of aggregated scHi-C matrices from each method, that are in the gold standard set. The gold standard set is defined as the significant differentially interacting locus-pairs detected by diffHic [39] from the cell type specific bulk Hi-C data. **G.** Correlation of CHESS [40] scores, depicting differential interactions of the cell types, between bulk Hi-C and aggregated scHi-C from different methods. Sample sizes for each violin plot of **B**-**D**, **G** are n = 23 corresponding to 23 chromosomes investigated.
